# Identification of QTNs, QTN-by-environment interactions, and their candidate genes for salt tolerance related traits in soybean

**DOI:** 10.1186/s12870-024-05021-8

**Published:** 2024-04-23

**Authors:** Ying Chen, Xiu-Li Yue, Jian-Ying Feng, Xin Gong, Wen-Jie Zhang, Jian-Fang Zuo, Yuan-Ming Zhang

**Affiliations:** 1 College of Plant Science and Technology, Huazhong Agricultural University, Wuhan, China; 2 College of Agriculture, Nanjing Agricultural University, Nanjing, China; 3 Ningxia Academy of Agriculture and Forestry Sciences, Crop Research Institute, Yinchuan, Ningxia China; 4 State Key Laboratory of Subtropical Silviculture, Zhejiang A&F University, Lin’an, Hangzhou, China

**Keywords:** Soybean, Salinity tolerance, 3VmrMLM, Genome-wide association study, QTN, QTN-by-environment interaction

## Abstract

**Background:**

Salt stress significantly reduces soybean yield. To improve salt tolerance in soybean, it is important to mine the genes associated with salt tolerance traits.

**Results:**

Salt tolerance traits of 286 soybean accessions were measured four times between 2009 and 2015. The results were associated with 740,754 single nucleotide polymorphisms (SNPs) to identify quantitative trait nucleotides (QTNs) and QTN-by-environment interactions (QEIs) using three-variance-component multi-locus random-SNP-effect mixed linear model (3VmrMLM). As a result, eight salt tolerance genes (*GmCHX1*, *GsPRX9*, *Gm5PTase8*, *GmWRKY*, *GmCHX20a*, *GmNHX1*, *GmSK1*, and *GmLEA2-1*) near 179 significant and 79 suggested QTNs and two salt tolerance genes (*GmWRKY49* and *GmSK1*) near 45 significant and 14 suggested QEIs were associated with salt tolerance index traits in previous studies. Six candidate genes and three gene-by-environment interactions (GEIs) were predicted to be associated with these index traits. Analysis of four salt tolerance related traits under control and salt treatments revealed six genes associated with salt tolerance (*GmHDA13*, *GmPHO1*, *GmERF5*, *GmNAC06*, *GmbZIP132*, and *GmHsp90s*) around 166 QEIs were verified in previous studies. Five candidate GEIs were confirmed to be associated with salt stress by at least one haplotype analysis. The elite molecular modules of seven candidate genes with selection signs were extracted from wild soybean, and these genes could be applied to soybean molecular breeding. Two of these genes, *Glyma06g04840* and *Glyma07g18150*, were confirmed by qRT-PCR and are expected to be key players in responding to salt stress.

**Conclusions:**

Around the QTNs and QEIs identified in this study, 16 known genes, 6 candidate genes, and 8 candidate GEIs were found to be associated with soybean salt tolerance, of which *Glyma07g18150* was further confirmed by qRT-PCR.

**Supplementary Information:**

The online version contains supplementary material available at 10.1186/s12870-024-05021-8.

## Background

Soil salinization is a major agricultural problem worldwide, especially in arid and semi-arid regions [1]. Salinity affects approximately 20% of irrigated cropland [2], resulting in a global loss of approximately 2,000 hectares of cropland per day. This contributes to a global annual loss of 1% to 2% of agricultural land [3, 4]. Higher soil salinity has negative effects on both soil properties and plant physiology [5].

Soybean (*Glycine max* L. Merr.) is a major source of edible vegetable oils and high-protein livestock feed [6, 7]. They are often considered to be more sensitive to salt stress than other crops [8]. Salt stress significantly reduces soybean yield, and high levels of salt damage the plant at all stages of the growth cycle. This includes germination, vegetative and reproductive growth, nodulation, leaf size, plant height, root length, shoot and root dry weight, seed size and seed weight [9–11]. Toxicity occurs when high concentrations of Cl^−^ and Na^+^ ions are absorbed and accumulated in the soybean plant. In previous studies, Na^+^ accumulation is more damaging to *Glycine soja*, while Cl^−^ accumulation is more damaging to *Glycine max* [12]. Exposure of soybean plants to salt stress resulted in reduction of hypocotyl and root length and fresh weight [13]. Root length, fresh root weight and dry root weight have been used as salinity tolerance indicators to evaluate the salinity tolerance of soybean [14].

Salt tolerance in plants is a complex quantitative trait that is influenced by numerous genetic and non-genetic factors [15, 16]. Quantitative trait locus (QTL) mapping and genome-wide association studies (GWAS) have been used as effective and precise tools to detect QTLs for salt tolerance-related traits, and a number of QTLs have been detected in previous studies. A total of 19 QTLs and 13 quantitative trait nucleotides (QTNs) for salt tolerance-related traits in soybean have been stored in Soybase (https://www.soybase.org/). In addition, Zeng et al. [17] identified 45 significant QTNs for salt tolerance-related traits in 283 different soybean accessions with 33,009 single nucleotide polymorphisms (SNPs) using GWAS. Shi et al. [18] identified 25 QTLs and 21 significant and 24 suggested QTNs for three salt tolerance indices in two environments. Cao et al. [14] associated salt tolerance-related traits at the seedling stage in 281 different soybean accessions with 58,112 SNPs, and 8, 4, 6 and 4 QTNs were found to be associated with germination ratio, root length, root fresh weight and root dry weight, respectively.

Currently, many salt tolerance-related genes in soybean have been reported to be involved in various salt tolerance mechanisms: ion transporters that maintain ion balance, such as *GmsSOS1* [19], *GmCHX1* [20], and *GmNHX1* [21]; osmotic adaptation, such as *GmWRKY27* [22]; restoration of oxidative balance, such as *GmPAP3* [23]; transcriptional regulation of salt stress responses, such as *GmNAC06* [24], *GmWRKY27* [22], *GmbZIP2* [25], *HSFB2b* [26], *GmMYB118* [27], *GmPHD* [28], *GmDREB6* [29], and *GmNFYA* [30].

To date, there are few GWAS reports on QTN-by-environment interactions (QEIs) for salt tolerance traits. In rice, Wang et al. [31] applied the QTLNetwork program to jointly analyze multi-environment datasets, and six, four and one QEIs were found to be associated with seedling height, shoot dry weight and root dry weight, respectively. In soybean, Zhang et al. [32] adopted the epistatic association mapping method of Lü et al. [33] to identify 83 QEIs for salt tolerance index. However, polygenic backgrounds are not included in the QTLNetwork program, and a limited number of markers were included in the model of Lü et al. [33], especially, their candidate genes, QEIs and gene-by-environment interactions (GEIs) are very limited. To address these issues, Li et al. [34] established a compressed variance component mixed model framework and the 3VmrMLM method to identify QTNs, QEIs and QTN-by-QTN interactions.

To address the above issues, the 3VmrMLM method was first used to detect QTNs and QEIs for salt tolerance-related traits in 286 soybean accessions. Then, candidate genes around significant and suggested QTNs and QEIs were mined using multi-omics methods. The study provides further understanding of the genetic structure of these traits and candidate genes and GEIs for soybean breeding and molecular biology studies.

## Materials and methods

### Genetic population

A total of 286 soybean accessions, including 14 wild, 153 landrace and 119 improved soybean accessions, were used in this study. These accessions were collected by the National Improvement Centre and the Linyi Academy of Agricultural Sciences and distributed in six geographical regions of China as described in our previous studies [32, 35]. 257 soybean accessions in 2009 and 2010 and 286 (additional 29) soybean accessions in 2014 and 2015 were planted in three-row plots in a randomized complete block design at the Jiangpu Experimental Station of Nanjing Agricultural University. The plot width was 1.5 m and the length was 2 m. When the seeds of each accession matured, they were harvested and used for the salt tolerance experiment.

### Phenotypes of four salt tolerance related traits in soybean accessions during germination stage

As described by Zhang et al. [32], the salt tolerance of all the 257/286 soybean accessions was evaluated using a salt water flooding method in the germination stage. Seeds of each accession were sown in a 30 × 20 × 15 cm plastic container with sand added to a height of 3.5 cm and treated with 350 ml water (CK, pH: 7.0) and 100 mM NaCl (pH: 7.0) solutions, respectively, with two replicates. Soybean seeds for each treatment were grown in a growth chamber under white fluorescent light (600 µmol m^−2^ s^−1^; 14 h light/10 h dark) at 25 ± 1 °C. Seven days after sowing, four salt-tolerance-related traits, including length of root (LR), dry weight of root (DWR), fresh weight of root (FWR), and length of hypocotyl (LH), were measured for each accession in the control and NaCl treatments in 2009, 2010, 2014, and 2015. Among these datasets, the phenotypic datasets in 2014 and 2015 are new, while the phenotypic datasets in 2009 and 2010 were reported in Zhang et al. [32].

To measure the degree of salt tolerance, the original trait observations were converted into a Salt Tolerance Index (STI) using the following equations [36]

$${\text{STI}}=\left({{\text{X}}}_{{\text{CK}}}-{{\text{X}}}_{{\text{NaC}}1}\right) / {{\text{X}}}_{{\text{CK}}}\times 100\%$$

where X_CK_ and X_NaCl_ were phenotypic values under control (CK) and saline (NaCl) treatments, respectively.

### Genotyping of soybean accessions

A total of 106,013 SNPs were obtained from Zhou et al. [35] by resequencing of 286 soybean accessions using the RAD-seq approach. To make the subsequent analysis results reliable, the SNPs with missing data ≥ 10% and a minimum allele frequency (MAF) ≤ 0.05 were filtered, and a total of 54,290 high-quality SNPs were obtained.

In addition, a total of 7,913,142 SNPs were obtained by resequencing 171 out of 286 soybean accessions. Similarly, SNPs with missing genotype rate ≥ 10% and MAF ≤ 0.03 were filtered, and 686,661 high quality SNPs were remained.

The above two genotypic datasets were merged and imputed using Beagle v5.2 software [37] with default settings, and a total of 740,754 high quality SNPs in 286 soybean accessions were obtained and used for this study.

### Statistical analysis for phenotypic traits

Phenotypic characteristics of four salt tolerance index traits were analyzed using the R package *psych*, including minimum, maximum, range, mean, standard deviation (SD), coefficient of variation (CV), kurtosis, and skewness. Correlation analysis between the four salt tolerance index traits was performed and visualized using the R package *GGally*. Two-way analysis of variance (ANOVA) was performed to determine the significance of genotypic and environmental variation using the R function *aov*. Best linear unbiased prediction (BLUP) values for all the accessions were calculated using the R package *lme4*. The broad sense heritability (*h*^2^) for each trait was calculated using the following equation

$${h}^{2}=\frac{{V}_{g}}{{V}_{g}+{V}_{e}/Ne}$$

where V_g_ was genetic variance, V_e_ was residual error variance, and *Ne* was the number of environments.

### Genome-wide association studies

The IIIVmrMLM software [38] of the 3VmrMLM method [34] was used to identify QTNs and QEIs for salt tolerance-related traits. In detail, the SingleEnv module was used to analyze each salt tolerance-related index trait in each environment for identifying QTNs, while the MultiEnv module was used to analyze each salt tolerance-related index trait in four environments and each trait (LR, LH, DWR, and FWR) between control and salt treatments for identifying QTNs and QEIs. The genotypes were the above 740,754 high quality SNPs from 286 soybean accessions. The kinship matrix K was calculated using IIIVmrMLM software. The number of optimal subgroups was calculated using ADMIXTURE [39]. The critical *P* value for significant QTNs and QEIs was set at 0.05/*m*, where *m* is the number of markers, while the critical LOD score for suggested QTNs and QEIs was set at 3.0 [34].

### Mining potential candidate genes for salt tolerance index traits in soybean

All the genes within the range of 150 kb downstream and upstream of each QTN for four salt tolerance-related traits were obtained from the soybean Glyma v1.1 genome annotation (glyma.Wm82.gnm1.ann1.DvBy.gene_models_main.gff3.gz), downloaded from Soybase (https://soybase.org/data/public/Glycine_max/). Among these genes, candidate genes for four salt tolerance-related traits were identified using comparative genomic analysis, gene differential expression analysis, KEGG pathway analysis, and soybean gene annotation. The details were as follows:

First, potential candidate genes whose gene annotations in soybean were related to salt stress responses were retained, where soybean gene annotation files were downloaded from both Phytozome12 (https://phytozome.jgi.doe.gov/pz/portal.html) and Soybase (https://www.soybase.org/). Second, the genes homologous to the *Arabidopsis* salt stress genes were retained. Then, potential candidate genes with KEGG pathway analysis involved in salt stress responses were retained using BlastKOALA version 2.2 [40]. Finally, the genes showing significant differential expression between control and salt treatments (log|FC|> 1.5; *P* < 0.05) were retained, with RNA-seq data downloaded from the NCBI GEO database (GEO accession ID: PRJNA766706) [41].

### SNP variants and haplotype analysis

SNP variants within and 2 kb upstream of the candidate genes were mined from the above genotypes. The genome sequences (glyma.Wm82.gnm1.FCtY.genome_main.fna.gz) and annotation (glyma.Wm82.gnm1.ann1.DvBy.gene_models_main.gff3.gz) were downloaded from Soybase (https://soybase.org/data/public/Glycine_max/) and used for SNP annotation using SnpEff software [42]. The SNP variants were extracted from the SnpEff annotated VCF file using a Perl script. We retained the loss-of-function mutations described by Torkamaneh et al. [43] and the variants in the 5’UTR, 3’UTR, and upstream of the candidate genes.

Haplotype analysis was performed using Haploview v4.1 software [44]. Based on the above phenotypes of the four traits, multiple comparisons of trait differences between different haplotypes were tested using the LSD.test function of the agricolae package in R.

### Co-expressional network analysis

The expression datasets of soybean genes under control and salt stress conditions in Li et al. [30], Sun et al. [45], and Lu et al. [46] were downloaded from the GEO database, and the GEO accessions were GSE93322 [30], GSE133574 [45], and GSE173640 [46], respectively. The transcript datasets from Lu et al. [46] included the counts of 24 samples, the leaf and root of transgenic plants and JACK plants under control and salt stress conditions with three replicates. The counts were converted to FPKM using the following equation

$$FPKM=\frac{{\text{C}}}{{\text{L}}\times {\text{N}}}\times {10}^{9}$$

where C was the count of each gene, L was the length of each gene’s CDS, and N was sum of all the gene counts.

The three transcript datasets were analyzed using the R package WGCNA v1.70 [47] to construct co-expression networks. Optimal soft thresholds were calculated using the function “pickSoftThreshold”, and the thresholds were set to r^2^ > 0.85. The TOMType and corType were set to “unsigned” and “bicor”, respectively. minModuleSize was set to 30, and mergeCutHeight was set to 0.3. The top 15 genes with higher kWithin value calculated by the intramodularConnectivity function of the WGCNA software were defined as hub nodes. The network was visualized using the Cytoscape package [48]. The KEGG enrichment analysis for the genes in the above co-expression networks was performed using the R package KOBAS [49].

### qRT-PCR verification of candidate genes

Plants were grown in a growth chamber under 16h light/8h dark (26°C). 7-d-old seedlings were exposed to either 200 mM NaCl or water as treatment and control, respectively. Roots were harvested at 0 and 6 h. Harvested samples were snap frozen in liquid nitrogen and stored at -80 °C for the following quantitative real-time PCR (qRT-PCR).

Total RNA was extracted from the samples using TRIzol reagent and quantified using a Nanodrop. The cDNA was synthesised using an EasyScript® One-Step gDNA Removal and cDNA Synthesis SuperMix (AE311, Transgen). The complete sequence information of the selected candidate genes was obtained from Phytozome v13 (https://phytozome-next.jgi.doe.gov/), and the corresponding primers are shown in Table S1 and were synthesised by Shenggong Bioengineering (Shanghai) Co., Ltd. Quantitative PCR was performed to amplify cDNA using 2X Universal SYBR Green Fast qPCR Mix (RK21203, ABclonal) and was performed on the BIO-RAD CFX Connect Real-Time PCR Detection System. The *actin11* gene was selected as an internal control to normalize the expression data. The 2^−ΔΔCt^ method was used to calculate the relative expression of genes. Each sample contains three replicates.

## Results

### Phenotypic variation of four salt tolerance index related traits

**Fig. 1:**
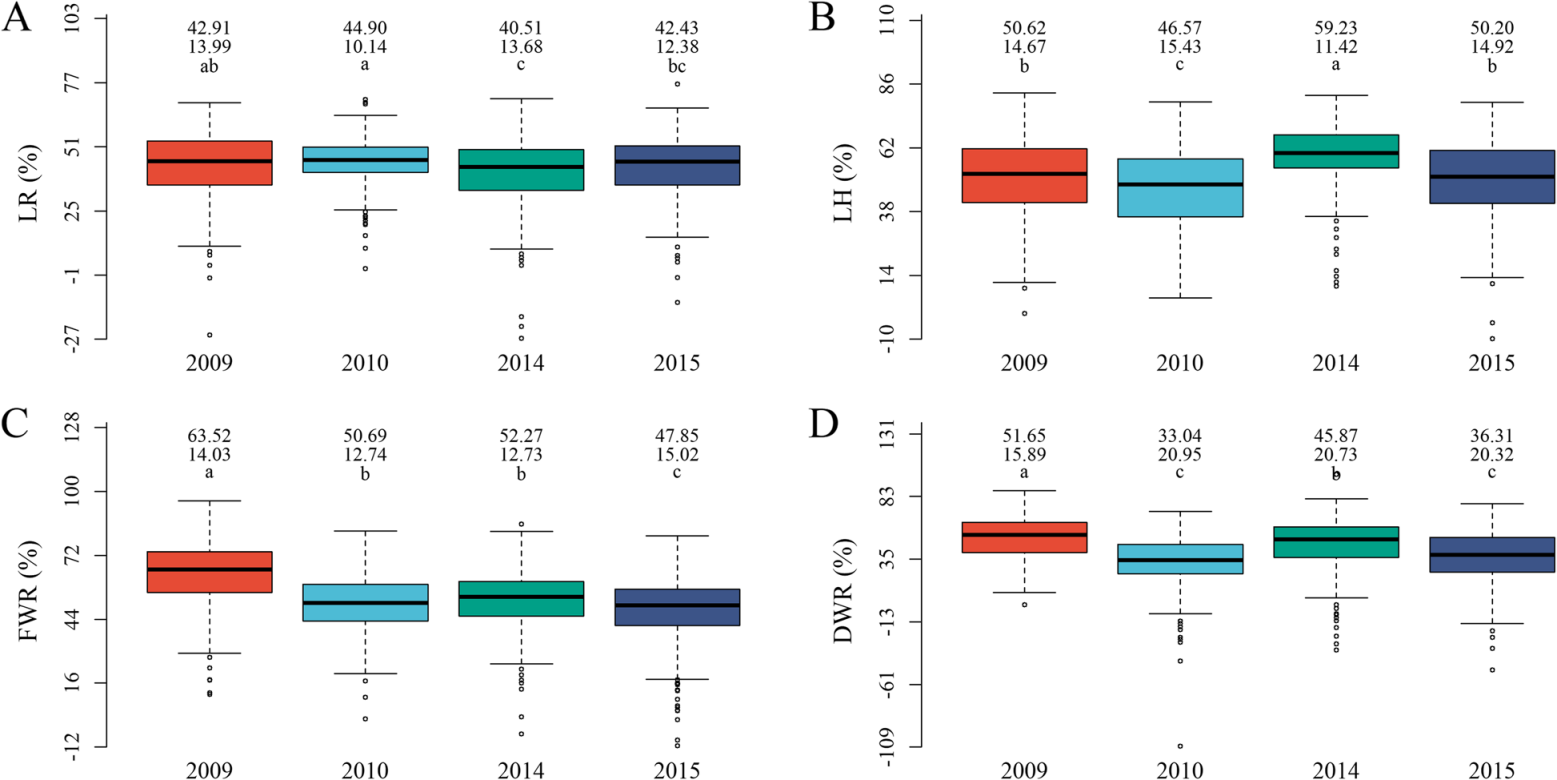
The boxplot of soybean salt tolerance index traits in four environments. **A**-**D** were the phenotype boxplots for LR, LH, FWR, and DWR in four environments; The first, second and three rows in the upper of each plot were the mean, standard deviation and multiple comparison results, respectively. The characters a-c in each boxplot marked the significance of these traits across different environments using multiple comparison

As described by Zhang et al. [32], LR, DWR, FWR, and LH were measured in 286 soybean accessions under control and salt treatments in 2009, 2010, 2014, and 2015, their salt tolerance indices were calculated and their traits are listed in Table S2. The coefficients of variation for these index traits and their best linear unbiased prediction (BLUP) values ranged from 8.12% to 63.4% with a mean of 28.60%, while their heritabilities ranged from 47.46% to 64.74% with a mean of 54.56% (Table S2). The five accessions with the minimum index (WenFeng 6, 84 Tie 0066, ZYD4157, ZYD4368, and Y117249) are listed as salt-tolerant accessions, while the five accessions with the maximum index (Kaifeng 80–7 Zao, Ludou 2, Riben Daheidou, He 05–47, and Nannong Heizhenzhu) are listed as salt-sensitive accessions. The phenotypic differences of these traits were significant across environments (Fig. 1). In two-way (genotype and environment) ANOVA, the genotypic variations for the four index traits were highly significant (*P*-value = 8.57e-29 ~ 3.62e-11) (Table S3), indicating the feasibility of conducting GWAS for the four index traits.

Correlation analysis of these index traits revealed significant positive correlations between the BLUP values of the four index traits, such as r^2^ = 0.736 between DWR and FWR (*P*-value < 0.001; Figure S1).

### Identification of QTNs for four salt tolerance index traits using 3VmrMLM

#### QTNs for four salt tolerance index traits using a single environment analysis

**Fig. 2:**
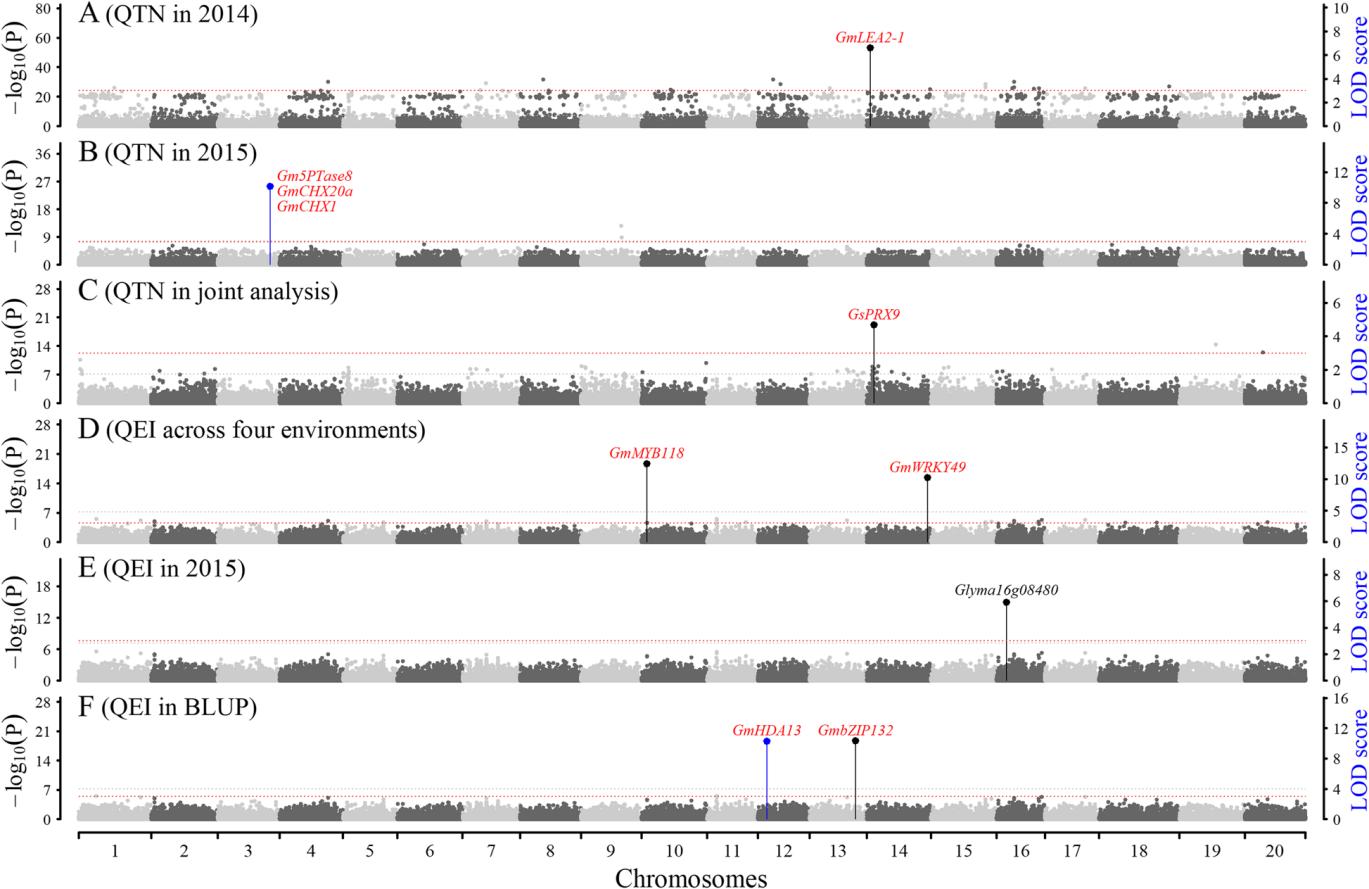
The Manhattan plot for length of main root (LR) using IIIVmrMLM software. **A**-**B**: QTNs for LR in 2014 and 2015 using single environment analysis of IIIVmrMLM. **C**: QTNs for LR using multi-environment joint analysis of IIIVmrMLM. **D**: QTN-by-environment interactions (QEIs) for LR using multi-environment joint analysis of IIIVmrMLM. **E**–**F**: QEIs for LR between control and salt treatments using multi-environment joint analysis of IIIVmrMLM. The black (one) and blue (multiple) lines indicate the times that the QTN/QEI was identified. The known and candidate genes were marked with red and black colors, respectively

**Fig. 3:**
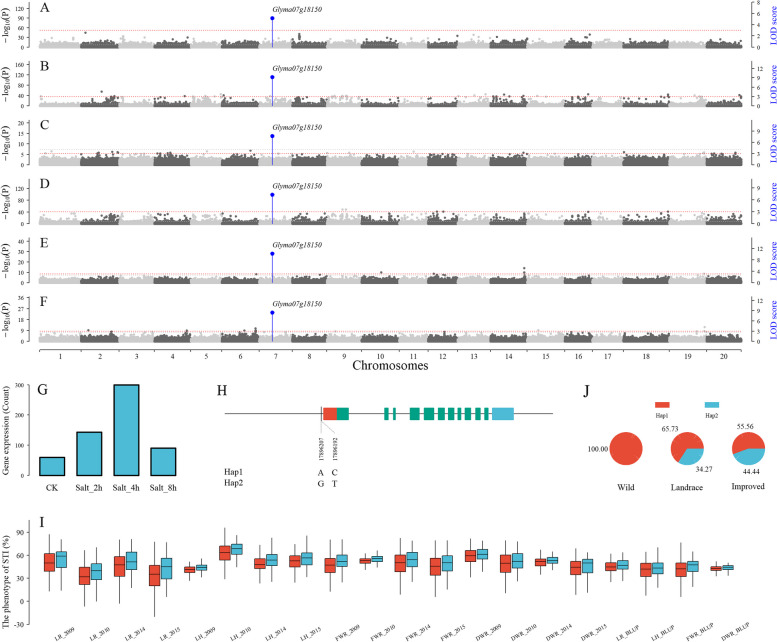
The candidate gene *Glyma07g18150* around QTN for salt tolerance index traits. **A**-**B**: Manhattan plot of QTNs for DWR in 2014 and BLUP values. **C**-**D**: Manhattan plot of QTNs for FWR in 2015 and BLUP values. **E**–**F**: Manhattan plot of QTNs for DWR and LH using multi-environment joint analysis. **G**. Differential expression levels (Count) of *Glyma07g18150* between under control and salt treatments. The gene expression levels were obtained from Hu et al. [41]. **H**: Two SNPs and their haplotypes of *Glyma07g18150*, in which 5'UTR and 3'UTR are marked by red and blue colors, respectively. **I**: Boxplot of salt tolerance index traits of two *Glyma07g18150* haplotypes in different environments. **J**: The haplotype frequencies of *Glyma07g18150* in wild, landrace, and bred soybeans

A total of 208 QTNs were identified on all the chromosomes for the above four salt tolerance index traits and their BLUP values (Figs. 2A-B, 3A-D, S2; Table S4). In detail, 33, 44, 30, and 42 significant QTNs were found to be associated with LR, LH, DWR, and FWR, respectively (*P*-value < 6.75e-8); their LOD scores were 7.16 ~ 80.61 for LR, 7.20 ~ 85.69 for LH, 7.21 ~ 143.73 for DWR, and 7.24 ~ 68.01 for FWR; the corresponding average r^2^ values were 4.33%, 3.80%, 4.53%, and 3.76%, respectively (Table S4).

15, 14, 13, and 22 suggested QTNs on all the chromosomes were found to be associated with LR, LH, DWR, and FWR, respectively (LOD > 3; Table S4); their LOD scores were 3.28 ~ 7.07 for LR, 3.05 ~ 7.08 for LH, 3.04 ~ 6.97 for DWR, and 3.19 ~ 6.71 for FWR; the corresponding average r^2^ values were 3.34%, 2.48%, 3.17%, and 2.44%, respectively (Table S4).

#### QTNs for salt tolerance index traits using multi-environment joint analysis

A total of 60 QTNs were detected on all the chromosomes for the above four salt tolerance index traits (Figs. 2C, 3E-F, S2; Table S5). In detail, 9, 13, 8, and 11 significant QTNs were found to be associated with LR, LH, DWR, and FWR, respectively (*P*-value < 6.75e-8). The LOD scores ranged from 8.10 to 21.98 for LR, 7.40 to 41.19 for LH, 7.75 to 34.60 for DWR, and 8.05 to 33.44 for FWR, and the corresponding average r^2^ values were 0.54%, 1.27%, 1.12%, and 0.72%, respectively, such as 2.55% for the LH QTN snp1466 (Table S5).

7, 3, 6, and 6 suggested QTNs on all the chromosomes were found to be associated with LR, LH, DWR, and FWR, respectively (Table S5). The LOD scores were 3.01 ~ 7.00 for LR, 4.14 ~ 6.82 for LH, 3.28 ~ 6.02 for DWR, and 4.94 ~ 5.77 for FWR, and the corresponding average r^2^ values were 0.29%, 0.65%, 0.77%, and 0.45%, respectively.

As mentioned above, a total of 258 QTNs were detected for the above four salt tolerance index traits using the single and multi-environment analyses of the 3VmrMLM method. Only 10 QTNs were shared between the two types of analyses.

### Identification of QEIs for the above four traits using 3VmrMLM

#### QEIs for the four index-related traits across environments

A total of 59 QEIs for salt tolerance index-related traits (iQEIs) were identified on all the chromosomes (Figs. 2D, S2; Table S6). In detail, 12, 9, 10, and 14 significant iQEIs were found to be associated with LR, LH, DWR, and FWR, respectively (*P*-values < 6.75e-8). The LOD scores ranged from 8.50 to 240.20 for LR, 8.18 to 101.72 for LH, 10.58 to 79.79 for DWR, and 9.61 to 143.89 for FWR, and the corresponding average r^2^ values were 6.09%, 4.97%, 4.61%, and 4.42%, respectively, such as 32.76% for the LR iQEI snp48170 (Table S6).

3, 4, 3, and 4 suggested iQEIs on all the chromosomes were found to be associated with LR, LH, DWR, and FWR, respectively (LOD > 3; Table S6). The LOD scores were 5.38 ~ 7.15 for LR, 5.83 ~ 9.26 for LH, 4.07 ~ 7.77 for DWR, and 5.16 ~ 8.26 for FWR, and the corresponding average r^2^ values were 0.51%, 0.98%, 1.01%, and 0.67%, respectively.

#### QEIs for the four traits between control and salt treatments

The trait observations and their BLUP values for the four traits in the control and salt treatments were used to identify QTNs and QEIs. A total of 166 QEIs on all the chromosomes were identified to be associated with the four traits (Figs. 2E-F, S2; Table S7). Among these QEIs, 28, 42, 21, and 25 significant QEIs were found to be associated with LR, LH, DWR, and FWR, respectively (*P*-value < 6.75e-8). The LOD scores were 6.61 ~ 43.14 for LR, 6.88 ~ 81.02 for LH, 6.34 ~ 306.87 for DWR, and 7.58 ~ 36.96 for FWR; the corresponding average r^2^ values were 1.48%, 1.02%, 7.06%, and 1.18%, respectively; there were four large QEIs (r^2^ > 10%), such as 69.86% for the DWR QEI Gm04:10,966,335 (Table S7).

14, 22, 9, and 11 suggested QEIs on all the chromosomes were found to be associated with LR, LH, DWR, and FWR, respectively (LOD > 3; Table S7). The LOD scores ranged from 4.36 to 7.12 for LR, 3.47 to 7.00 for LH, 3.02 to 6.59 for DWR, and 3.03 to 6.58 for FWR, and the corresponding average r^2^ values were 0.52%, 0.32%, 0.48%, and 0.40%, respectively.

There was no common QEI for salt tolerance index-related traits and salt tolerance-related traits between control and salt treatments.

### Mining known and candidate genes around all the QTNs for the four salt tolerance index traits

#### Known salt tolerance genes

Within the 150 kb flanking genomic region for each QTN for the four salt tolerance index traits, there were 4646 genes. Of these, eight genes were shown to be associated with salt stress in previous studies (Table 1), including *GmCHX1* [20], *GsPRX9* [50], *Gm5PTase8* [51], *GmWRKY* [52], *GmCHX20a* [53], *GmNHX1* [21], *GmSK1* [54], and *GmLEA2-1* [55] (Table 1).

**Table 1:** Eight known and six candidate genes around QTNs for salt tolerance index traits in soybean

Trait	Dataset	Marker	Chr	Pos (bp)	LOD	Add	Dom	r^2^(%)	*P*-value	Sign	Gene	Homologous gene in Arabidopsis			Reference
												**Gene**	**Symbol**	**Annotation**	
DWR	II	Gm11:6,095,319	11	6,095,319	6.97	5.7	-0.91	1.54	1.06E-07	SUG	*Glyma11g08440*(*GmSK1*)	AT1G75950	SKP1	S phase kinase-associated protein 1	Chen et al. [54]
DWR	I	snp67755	14	2,766,500	8.01	4.29	2.30	2.61	9.71E-09	SIG	*Glyma14g04180*(*GmLEA2-1*)	AT2G44060		Late embryogenesis abundant protein, group 2	Wang et al. [55]
DWR	V	snp68284	14	5,836,612	12.85	1.95	1.38	2.62	1.43E-13	SIG	*Glyma14g07730*(*GsPRX9*)	AT1G44970		Peroxidase superfamily protein	Jin et al. [50]
FWR	I	snp50832	10	38,837,251	4.79	-2.29	0.55	2.03	1.62E-05	SUG	*Glyma10g30020*(*GmNHX1*)	AT3G05030	NHX2	sodium hydrogen exchanger 2	Li et al. [21]
FWR	I	Gm14:47,141,962	14	47,141,962	16.19	4.30		2.27	5.94E-18	SIG	*Glyma14g38010*(*GmWRKY49*)	AT2G38470	WRKY33	WRKY DNA-binding protein 33	Xu et al. [56]
LR	IV	Gm03:40,724,131	3	40,724,131	10.35	2.96		1.45	5.02E-12	SIG	*Glyma03g32890*(*GmCHX20a*)	AT3G53720	CHX20	cation/H + exchanger 20	Jia et al. [53]
LR	IV	Gm03:40,724,131	3	40,724,131	10.35	2.96		1.45	5.02E-12	SIG	*Glyma03g32900*(*GmCHX1*)	AT3G53720	CHX20	cation/H + exchanger 20	Qi et al. [20]
LR	IV	Gm03:40,724,131	3	40,724,131	10.35	2.96		1.45	5.02E-12	SIG	*Glyma03g33035*(*Gm5PTase8*)	AT2G37440		DNAse I-like superfamily protein	Jia et al. [51]
LR	III	snp67772	14	2,907,230	6.58	0.17	-14.79	4.62	2.63E-07	SUG	*Glyma14g04180*(*GmLEA2-1*)	AT2G44060		Late embryogenesis abundant protein, group 2	Wang et al. [55]
LR	VI	snp68268	14	5,726,780	4.66	-1.22	1.16	0.37	2.18E-05	SUG	*Glyma14g07730*(*GsPRX9*)	AT1G44970		Peroxidase superfamily protein	Jin et al. [50]
DWR	II	Gm06:3,502,387	6	3,502,387	143.73	49.29	39.77	9.39	1.89E-144	SIG	*Glyma06g04840*	AT1G27730	STZ	salt tolerance zinc finger	
DWR	III	snp34353	7	17,960,593	5.13	-3.92	8.84	2.44	7.43E-06	SUG	*Glyma07g18150*	AT3G51860	CAX3	cation exchanger 3	
DWR	V	snp34353	7	17,960,593	9.21	-1.64	0.46	3.75	6.13E-10	SIG	*Glyma07g18150*	AT3G51860	CAX3	cation exchanger 3	
DWR	VI	snp46475	9	39,073,956	10.27	-3.20	0.65	1.42	5.33E-11	SIG	*Glyma09g32570*	AT4G29080	PAP2	phytochrome-associated protein 2	
FWR	V	snp11062	3	1,445,425	9.66	-0.93	3.46	2.22	2.17E-10	SIG	*Glyma03g01603*	AT4G00630	KEA2	K + efflux antiporter 2	
FWR	VI	snp11062	3	1,445,425	12.68	-2.16	0.20	0.47	2.08E-13	SIG	*Glyma03g01603*	AT4G00630	KEA2	K + efflux antiporter 2	
FWR	IV	Gm07:17,751,732	7	17,751,732	7.67	0.23	-9.31	3.29	2.15E-08	SIG	*Glyma07g18150*	AT3G51860	CAX3	cation exchanger 3	
FWR	V	snp34353	7	17,960,593	7.24	-0.85	-0.08	1.63	5.69E-08	SIG	*Glyma07g18150*	AT3G51860	CAX3	cation exchanger 3	
FWR	VI	snp34353	7	17,960,593	10.47	-1.97	1.35	0.53	3.40E-11	SIG	*Glyma07g18150*	AT3G51860	CAX3	cation exchanger 3	
FWR	II	snp88309	18	2,040,047	6.22	3.39	-6.18	6.53	5.98E-07	SUG	*Glyma18g03090*	AT3G08510	PLC2	phospholipase C 2	
LH	VI	snp34355	7	17,965,274	8.41	0.87	-7.28	1.1	3.86E-09	SIG	*Glyma07g18150*	AT3G51860	CAX3	cation exchanger 3	
LH	I	Gm18:2,022,673	18	2,022,673	22.13	4.12	9.70	5.77	7.46E-23	SIG	*Glyma18g03090*	AT3G08510	PLC2	phospholipase C 2	
LH	V	snp100307	19	47,249,031	7.76	1.34	-1.51	4.53	1.76E-08	SIG	*Glyma19g40980*	AT1G09530	PIF3	phytochrome interacting factor 3	

*DWR* the dry weights of roots, *FWR* the fresh weights of roots, *LR* the length of main root, *LH* the length of hypocotyls, Dataset I ~ V: the detection of main-effect QTNs for the phenotype of salt tolerance index traits in 2009, 2010, 2014, 2015, and BLUP using Single-Env method of 3VmrMLM; Dataset VI: the detection of main-effect QTNs for the phenotype of salt tolerance index traits across all environment using Multi-Env method of 3VmrMLM; *Chr* chromosome, *Pos* position, *Add* additive effect, *Dom* dominance effect, *SIG* significant, *SUG* suggestion

### Candidate salt tolerance genes

Based on comparative genomic analysis, KEGG analysis, and differentially expressed analysis, the above 4638 genes were used to mine candidate salt tolerance genes.

According to the previously reported soybean salt tolerance mechanisms and gene functional annotations, 19 candidate genes were found to be related to salt stress. Based on homology analysis, 14 genes were homologous to *Arabidopsis thaliana* genes that were reported to be related to salt stress in previous studies. The remaining genes were subjected to KEGG analysis. As a result, 118 genes were involved in the pathways associated with salt stress responses, including ABC transporters, arginine and proline metabolism, MAPK signaling pathway, phytohormone signaling and sulfur metabolism. Thus, 151 genes were predicted to be associated with salt stress responses. To further confirm these potential candidate genes, differential expression analysis was performed using the RNA-seq data of Hu et al. [41]. As a result, 54 genes showed significant differential expression levels between control and salt stress treatments, such as the candidate gene *Glyma07g18150* (Fig. 3G).

The SNP genotypes of 286 soybean accessions were used to identify SNP variants within candidate genes and their 2 Kb upstream sequences. As a result, 17 out of the above 54 genes had SNP variants. In particular, six SNP variants of *Glyma09g35300* and one SNP variant of *Glyma19g40980* were missense variants. The SNP variants of the 17 genes were used for haplotype analysis. Among 14 genes identified in single environment analysis, five genes (*Glyma06g04840*, *Glyma03g01603*, *Glyma07g18150*, *Glyma18g03090*, and *Glyma19g40980*) showed significant differences in salt tolerance index traits among different haplotypes in one-way ANOVA (Table 1). Among 6 genes identified in the joint analysis of all environments, three genes (*Glyma03g01603*, *Glyma07g18150*, and *Glyma09g32570*) showed significant differences in salt tolerance index traits across different haplotypes in two-way (haplotype and year) ANOVA (Table 1), such as candidate gene *Glyma07g18150* (Figs. 3H-I). In conclusion, eight candidate genes associated with the four index traits were identified.

In addition, *Glyma06g04840* and *Glyma07g18150* were considered as important candidate genes, and qRT-PCR verification experiment was conducted. The relative expression levels of *Glyma06g04840* and *Glyma07g18150* in roots were significantly higher in 6 h salt treatment than in control (Figure S3), indicating the involvement of these genes in salt stress response.

### Mining known and candidate GEIs for the four salt tolerance index traits

#### Known genes of salt tolerance index traits in multi-environment analysis

Within the 150 kb flanking genomic region for each iQEI, there were a total of 1147 genes. Among these genes, two genes, including *GmSK1* [54] and *GmWRKY49* [56], were verified to regulate soybean salt tolerance index traits (Table 2).

**Table 2:** Two known and three candidate genes around QEIs for salt tolerance index traits in soybean

Trait	Marker	Chr	Pos (bp)	LOD	r^2^(%)	*P*-value	Sign	Gene	Homologous gene in Arabidopsis			Reference
									**Gene**	**Symbol**	**Annotation**	
DWR	Gm11:6,095,319	11	6,095,319	79.79	15.32	2.76E-76	SIG	*Glyma11g08440*(*GmSK1*)	*AT1G75950*	SKP1	S phase kinase-associated protein 1	Chen et al. [54]
LR	Gm14:47,141,670	14	47,141,670	22.58	1.88	2.17E-22	SIG	*Glyma14g38010*(*GmWRKY49*)	*AT2G38470*	WRKY33	WRKY DNA-binding protein 33	Xu et al. [56]
DWR	snp20887	4	47,545,085	10.5766	1.7005	8.54E-09	SIG	*Glyma04g41701*	*AT5G24110*	WRKY30	WRKY DNA-binding protein 30	
LH	Gm13:35,351,832	13	35,351,832	13.5898	1.9055	1.65E-13	SIG	*Glyma13g33590*	*AT1G53580*	GLY3	glyoxalase II 3	
FWR	Gm16:26,218,707	16	26,218,707	26.1218	2.9313	1.42E-23	SIG	*Glyma16g22630*	*AT4G35640*	SERAT3;2	serine acetyltransferase 3;2	

*DWR* the dry weights of roots, *FWR* the fresh weights of roots, *LR* the length of main root, *LH* the length of hypocotyls, *Chr* chromosome, *Pos* position, *SIG* significant, *SUG* suggestion

#### Candidate GEIs of salt tolerance index traits in multi-environment analysis

The above 1145 genes were used to mine candidate salt tolerance GEIs as described below.

According to the previously reported soybean salt tolerance mechanisms and gene functional annotations, 12 potential candidate salt stress-related genes were identified. Based on homology analysis, five genes were homologous to *Arabidopsis thaliana* genes reported to be responsible for salt stress in previous studies. The residues were used for KEGG pathway analysis. 16 genes were predicted to be involved in the pathways associated with salt stress responses, including ABC transporters, arginine and proline metabolism, MAPK signaling pathway, and sulfur metabolism. Thus, 33 genes were found to be potentially associated with salt stress responses. To further confirm these genes, differential expression analysis was performed using RNA-seq data from Hu et al. [41], and seven genes showed significantly different expression levels between control and salt stress treatments.

The genotypes of 286 soybean accessions were used to identify the SNP variants within these potential candidate genes and their 2 Kb upstream sequences. A total of 6 potential candidate genes were found to have SNP variants. In particular, two SNPs of *Glyma12g07270* and three SNPs of *Glyma13g33590* were missense variants. The SNP variants in all of 6 potential candidate genes were used for haplotype analysis, and three genes (*Glyma04g41701*, *Glyma13g33590*, and *Glyma16g22630*) showed significant differences in salt tolerance index traits in haplotype-by-environment interactions (Table 2).

The promoter sequences of the three genes were used to identify their cis-acting elements. All of the three genes had multiple cis-acting elements involved in environmental responses, including cis-acting regulatory elements involved in MeJA responsiveness, abscisic acid responsiveness, auxin responsiveness, light responsiveness, drought inducibility, defence and stress responsiveness, low temperature responsiveness, salicylic acid responsiveness, and gibberellin responsiveness. These results further indicated that these candidate genes regulate salt-tolerance-related traits and respond to environmental variations.

### Mining known and candidate GEIs around QEIs for four salt tolerance related traits between control and salt treatments

#### Known genes in the analysis of salt tolerance related traits

Within the 150 kb flanking genomic region for each QEI for salt tolerance index related traits, there were a total of 3148 genes. Among these genes, six genes, including *GmHDA13* [30], *GmPHO1* [57], *GmERF5* [58], *GmNAC06* [24], *GmbZIP132* [59], and *GmHsp90s* [60], were verified to regulate salt-tolerance-related traits in soybean (Table 3).

**Table 3:** Six known and five candidate genes around QEIs for salt tolerance index traits in soybean

Traits	Dataset	Marker	Chr	Pos (bp)	LOD	r^2^	*P* value	Signi	Gene	Homologous gene in Arabidopsis			Reference
										**Gene**	**Symbol**	**Annotation**	
LH	I	snp5442	2	394,740	4.04	0.32	9.16E-05	SUG	*Glyma02g00640*(*GmPHO1*)	*AT3G23430*	PHO1	phosphate 1	Wang et al. [57]
LH	IV	Gm09:3,553,566	9	3,553,566	7.54	0.43	2.86E-08	SIG	*Glyma09g04630*(*GmERF5*)	*AT3G16770*	EBP	ethylene-responsive element binding protein	Dong et al. [58]
LH	II	snp57179	12	1,726,812	8.13	0.79	7.37E-09	SIG	*Glyma12g02540*(*GmNAC06*)	*AT2G17040*	NAC036	NAC domain containing protein 36	Li et al. [24]
LH	IV	snp57993	12	7,068,862	5.67	0.32	2.14E-06	SUG	*Glyma12g09190*(*GmHDA13*)	*AT3G44680*	HDA9	histone deacetylase 9	Lu et al. [30]
LH	IV	snp67491	14	937,675	19.75	1.18	1.79E-20	SIG	*Glyma14g01530*(*GmHsp90s*)	*AT5G56000*	Hsp81.4	HEAT SHOCK PROTEIN 81.4	Xu et al. [60]
LR	V	snp57993	12	7,068,862	27.51	0.81	3.11E-28	SIG	*Glyma12g09190*(*GmHDA13*)	*AT3G44680*	HDA9	histone deacetylase 9	Lu et al. [30]
LR	V	snp66073	13	35,982,335	29.88	0.89	1.33E-30	SIG	*Glyma13g34460*(*GmbZIP132*)	*AT3G14880*		unknown	Liao et al. [59]
LH	IV	snp9763	2	44,325,265	4.51	0.25	3.12E-05	SUG	*Glyma02g38910*	*AT5G58940*	CRCK1	calmodulin-binding receptor-like cytoplasmic kinase 1	
LH	V	snp57235	12	2,202,736	3.95	0.09	1.13E-04	SUG	*Glyma12g03200*	*AT3G19830*	NTMC2TYPE5.2	Calcium-dependent lipid-binding (CaLB domain) family protein	
LH	IV	snp82823	16	31,773,546	16.76	0.99	1.74E-17	SIG	*Glyma16g27950*	*AT2G33710*		Integrase-type DNA-binding superfamily protein	
LH	IV	snp93385	18	52,687,266	4.06	0.23	8.71E-05	SUG	*Glyma18g43250*	*AT1G09950*	RAS1	RESPONSE TO ABA AND SALT 1	
LR	IV	snp80040	16	7,789,725	5.90	0.49	1.27E-06	SUG	*Glyma16g08480*	*AT3G28345*		ABC transporter family protein	

*LH* the length of hypocotyls, *LR* the length of main root, Dataset I ~ V: the detection of main-effect QTNs for the phenotype of salt tolerance related traits between control and salt treatment in 2009, 2010, 2014, 2015, and BLUP using Multi-Env method of 3VmrMLM, *Chr* chromosome, *Pos* position, *SIG* significant, *SUG* suggestion

#### Candidate salt tolerance GEIs

The above 3142 genes were used to mine candidate salt tolerance GEIs.

According to the previously reported soybean salt tolerance mechanisms and gene functional annotations, 58 genes were found to be related to salt stress. Based on homology analysis, three genes were found to be homologous to *Arabidopsis thaliana* genes reported to be involved in salt stress in previous studies. The remains were used for KEGG pathway analysis, and 21 genes were found to be involved in the pathways of salt stress responses, including ABC transporters, arginine and proline metabolism, MAPK signaling pathway, and sulfur metabolism. Thus, all the 82 genes were found to be potentially associated with salt stress responses. To further confirm these genes, differential expression analysis was performed using the RNA-seq data from Hu et al. [41], and 45 genes showed significant differential expression levels between control and salt stress treatments.

The genotypes of 286 soybean accessions were used to search for SNP variants within these genes and their 2 Kb upstream sequences. A total of fifteen genes had SNP variants. In particular, one SNP of *Glyma04g09550* was a missense variant. The SNP variants of the fifteen genes were used for haplotype analysis, and five genes (*Glyma02g38910*, *Glyma12g03200*, *Glyma16g08480*, *Glyma16g27950*, and *Glyma18g43250*) showed significant differences in salt tolerance index traits in the haplotype-by-environment interactions (Table 3).

The promoter sequence of the above five genes was used to identify cis-acting elements of these genes, and all of the five genes had cis-acting elements involved in environmental responses, such as cis-acting elements involved in light responsiveness, MeJA responsiveness, abscisic acid responsiveness, drought inducibility, gibberellin responsiveness, salicylic acid responsiveness, auxin responsiveness, and low temperature responsiveness. In conclusion, the five GEIs regulated the salinity tolerance index traits and responded to the environment.

## Discussion

Soybean provides 59% of the world's oilseed production and 69% of the daily vegetable protein consumed [61]. Global soybean production must increase substantially to meet the world's rapidly growing food demand [30]. However, soybean yield is seriously threatened by unfavorable environmental factors. Genes associated with salt stress tolerance could be used to breed new soybean varieties with high salt tolerance [41]. Although some genes have been reported to regulate salt tolerance-related traits under these conditions [27, 54–56, 62], few QEIs and GEIs have been reported due to the limitations of QEI detection methods in GWAS. Note that QTNs not affected by different environments are identified from a single dataset or multiple environment datasets, while QEIs affected by different environments are identified only from multiple environment datasets. Recently, our group established a new comprehensive GWAS method, 3VmrMLM, to detect QTNs, QEIs, and QTN-by-QTN interactions while controlling for all possible polygenic backgrounds [34, 38]. Therefore, this study focused on the identification of QTNs, QEIs, and their known and candidate genes in different environments. As a result, ten known salt tolerance genes and a major salt tolerance QTL on chromosome 3 reported in previous studies [20, 63] around 258 QTNs and 59 iQEIs, and 6 known salt tolerance genes around 166 QEIs identified between control and salt treatments were found, indicating the reliability of our results. Meanwhile, 6 candidate salt tolerance genes and 3 candidate salt tolerance GEIs around 258 QTNs and 59 iQEIs and 5 candidate salt tolerance GEIs around 166 QEIs were found. More importantly, candidate salt tolerance genes *Glyma06g04840* and *Glyma07g18150* were confirmed by qRT-PCR. These known and candidate genes provide gene sources for soybean breeding and molecular biology research.

### The co-expression network analysis of salt tolerance related genes

**Fig. 4:**
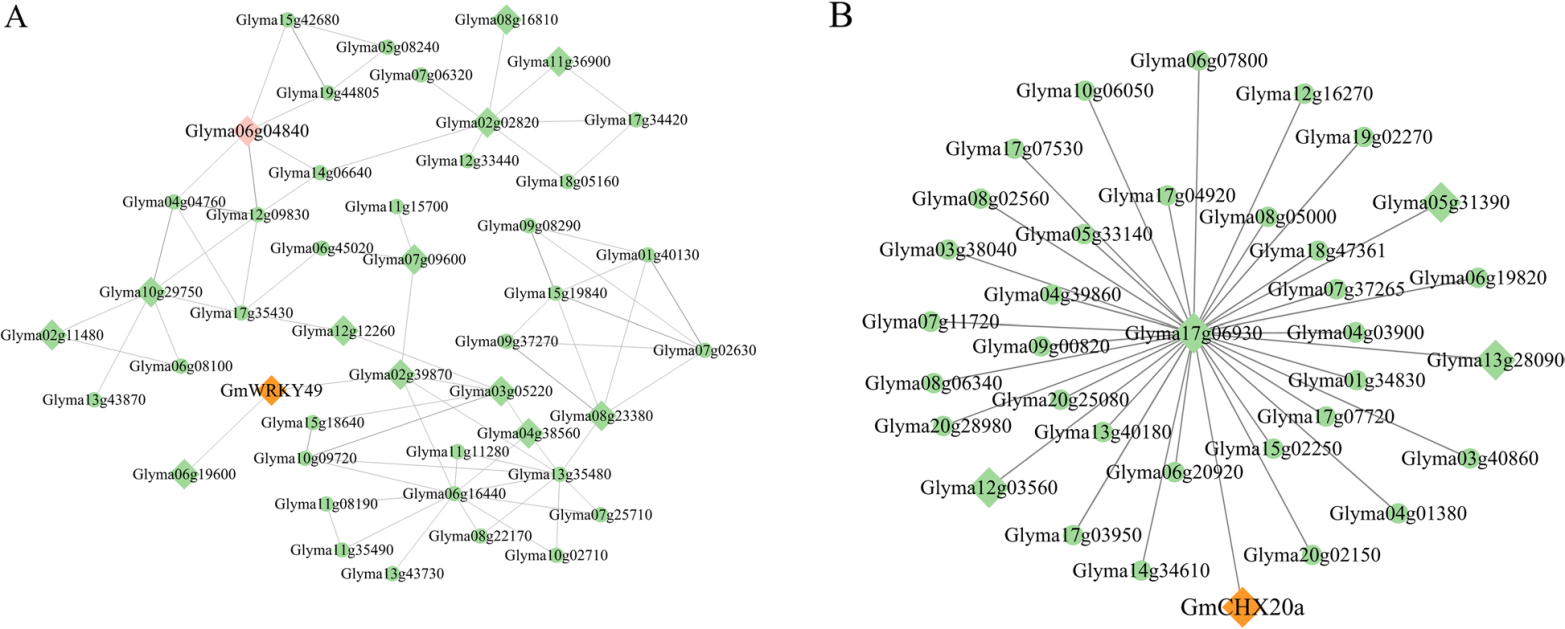
The subnetwork of the candidate and known genes. **A**: The magenta module; **B**: The turquoise module. The candidate and known genes were marked with pink and orange colors, respectively. The hub genes of each module were marked with diamond shape

To understand the co-expression network regulating salt tolerance traits, three transcript datasets from Li et al. [45], Sun et al. [46], and Lu et al. [30] were used in this study. A total of 1942 differential expression genes (DEGs) were identified using the R package DEGseq [64]. The expression levels of the above DEGs were then used to construct co-expression network using the R package WGCNA v1.70 [47]. As a result, 12 co-expression modules were constructed, including black (96), blue (319), brown (274), green (140), green-yellow (52), magenta (93), pink (93), purple (55), red (123), turquoise (385), and yellow (148) modules, where one, one, one and one known genes identified in this study were included in the purple, blue, magenta, and turquoise modules, respectively, and one and one candidate genes predicted in this study were included in the purple and magenta modules, respectively. The genes in each co-expression module were used to perform KEGG pathway enrichment analysis using KOBAS [49]. The results showed that the turquoise, magenta, blue, and purple modules were enriched in 24, 3, 2, and 2 KEGG pathways, respectively (corrected *P*-value < 0.05, Table S8). Among these pathways, 1, 2, 0 and 0 pathways were found to be associated with salt tolerance, including ‘plant hormone signaling’, ‘MAPK signaling pathway’, and ‘arginine and proline metabolism’ pathways (Table S8), which were reported to play an important role in the process of plant salt stress response in Liu et al. [65]. We analyzed the hub genes of the turquoise and magenta modules. The known gene *GmCHX20a* [53] in this study was the hub gene of the turquoise module, while the known gene *GmWRKY49* [56] and the candidate gene *Glyma06g04840* in this study were the hub genes of the magenta module (Fig. 4), indicating the co-expression of the three genes in this study with salt stress responses through the ‘plant hormone signaling’, ‘MAPK signaling pathway’, and ‘arginine and proline metabolism’ KEGG pathways.

### Domestication and improvement analyses of salt tolerance related candidate genes provided gene resource in future soybean breeding

Compared to wild soybeans, cultivated soybeans have lost a large number of important genes related to environmental adaptation during long-term domestication and improvement processes [66]. Wild relatives generally have a more diversified genomic pool and greater genetic variation than domesticated species and, providing breeders with a diverse range of genetic resources, including the genes for different stress tolerances [67].

**Fig. 5:**
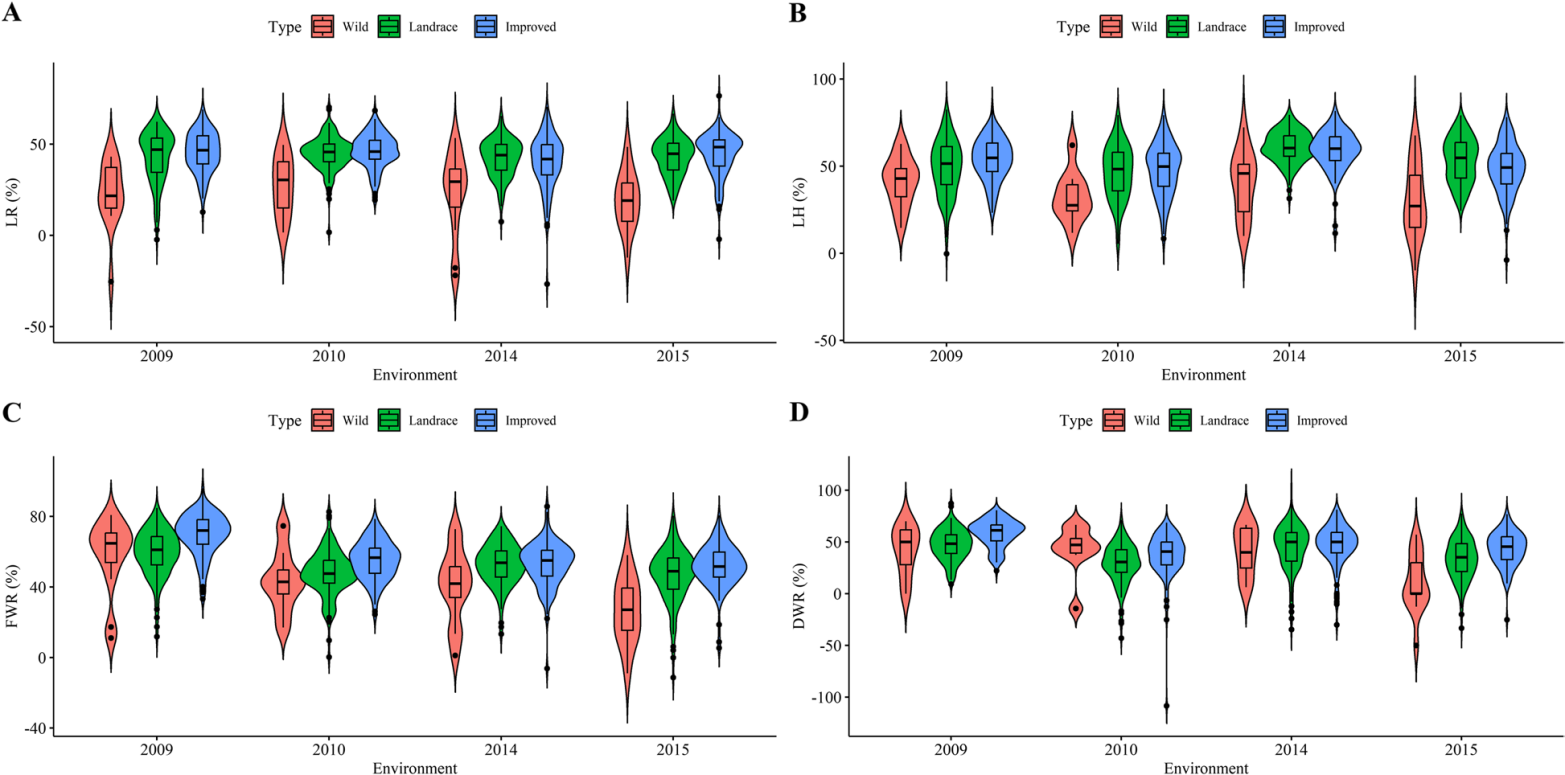
The salt tolerance index traits in wild, landrace, and improved soybean

In this study, the phenotypes of salt tolerance-related traits in wild soybean were significantly smaller than those in landrace and improved soybean (Table S9; Fig. 5), indicating greater salt tolerance of wild soybean than that of landrace and improved soybeans. This is consistent with a previous study [66]. Compared with the domestication and improvement regions of Zuo et al. [68], the known gene *GmNHX1* and two candidate genes (*Glyma19g40980* and *Glyma07g18150*) around QTNs for salt tolerance index traits and two candidate GEIs (*Glyma13g33590* and *Glyma04g41701*) for salt tolerance index traits were located in the domestication regions (Table 4). These genes may have undergone the domestication process. Two known genes (*GmNHX1* and *GmSK1*) and three candidate genes (*Glyma07g18150*, *Glyma18g03090*, and *Glyma03g01603*) around QTNs for salt tolerance index traits, and the known gene *GmSK1* and two candidate GEIs (*Glyma13g33590* and *Glyma16g22630*) for salt tolerance index traits, and three known genes (*GmHsp90s*, *GmbZIP132*, and *GmNAC06*) and one candidate GEI (*Glyma02g38910*) for salt tolerance related traits, were located in the improvement regions (Table 4). These genes may be undergoing an improvement process.

**Table 4:** The domestication and improvement of candidate genes in this study

	**Trait**	**Env**	**Gene**	***P*-value**		**Elite haplotype**	**Positions of haplotype bases**	**Frequencies of elite haplotype (%)**			**Selection region**	
				**Hap**	**Env**			**Wild**	**Landrace**	**Improved**	**Dom**	**Imp**
QTN	DWR	2010	*Glyma06g04840*	1.50E-03		AT; GC	3,428,833; 3,428,837	0.00	25.53	20.59		
	DWR	2014	*Glyma07g18150*	2.99E-03		CA	17,896,192; 17,896,207	100.00	68.18	56.73	dom287	imp150
	DWR	BLUP	*Glyma07g18150*	6.99E-06		CA	17,896,192; 17,896,207	100.00	65.73	55.56	dom287	imp150
	FWR	2010	*Glyma18g03090*	1.56E-05		GTG; GTA; TGA	2,036,717; 2,040,047; 2,040,057	85.71	76.43	59.26		imp463
	FWR	2015	*Glyma07g18150*	3.42E-04		CA	17,896,192; 17,896,207	100.00	65.47	56.19	dom287	imp150
	FWR	BLUP	*Glyma03g01603*	7.61E-03		GAG	1,383,737; 1,383,762; 1,395,406	22.22	0.00	0.00		imp43
	FWR	BLUP	*Glyma07g18150*	4.72E-06		CA	17,896,192; 17,896,207	100.00	65.73	55.56	dom287	imp150
	LH	BLUP	*Glyma19g40980*	1.90E-03		G	47,278,753	38.46	11.41	21.43	dom909	
	DWR	Joint	*Glyma09g32570*	3.58E-02	5.99E-09	C	39,084,436	91.49	90.09	91.65		
	FWR	Joint	*Glyma03g01603*	1.30E-04	4.54E-27	GAG	1,383,737; 1,383,762; 1,395,406	22.58	0.00	0.00		imp43
	FWR	Joint	*Glyma07g18150*	1.15E-07	1.33E-29	CA	17,896,192; 17,896,207	100.00	66.73	55.99	dom287	imp150
	LH	Joint	*Glyma07g18150*	1.06E-03	1.20E-02	CA	17,896,192; 17,896,207	100.00	66.48	55.99	dom287	imp150
iQEI	DWR		*Glyma04g41701*	1.68E-02	5.75E-07	A	47,556,222	48.48	17.61	27.72	dom163	
	FWR		*Glyma16g22630*	6.81E-03	7.42E-30	AT; GA	26,127,917; 26,128,002	40.48	2.77	0.00		imp425
	LH		*Glyma13g33590*	5.09E-03	5.31E-03	AATGT	35,390,245; 35,393,088; 35,393,093; 35,393,098; 35,393,100	9.30	0.00	0.89	dom613	imp338
QEI	LH	2015	*Glyma02g38910*	9.48E-03	1.93E-167	A	44,246,582	75.00	27.87	22.33		imp39
	LH	2015	*Glyma16g27950*	8.99E-04	6.40E-168	A	31,899,123	25.00	56.10	21.60		
	LH	2015	*Glyma18g43250*	2.56E-02	2.55E-129	AT	52,686,280; 52,687,266	8.33	12.79	5.35		
	LH	BLUP	*Glyma12g03200*	3.01E-09	5.88E-277	C	2,103,129	33.33	0.65	0.00		
	LR	2015	*Glyma16g08480*	3.49E-02	3.11E-117	A	7,888,926	45.45	14.33	16.29		

*DWR* the dry weights of roots, *FWR* the fresh weights of roots, *LH* the length of hypocotyls, *LR* the length of main root, *Env* environment, Selection region: the domestication and improvement regions in Zuo et al. (2022)

To further confirm these candidate genes and GEIs, their elite haplotype frequencies in wild, landrace, and improved soybean were calculated in 286 soybean accessions. As a result, a total of 7 candidate genes were further confirmed. During the domestication process, the elite haplotype frequencies of two candidate genes (*Glyma19g40980* and *Glyma07g18150*) around QTNs for salt tolerance index traits and two candidate GEIs (*Glyma13g33590* and *Glyma04g41701*) for salt tolerance index traits were higher in wild soybean than in landrace soybean (Table 4). During the improvement process, the elite haplotype frequencies of two candidate genes (*Glyma07g18150* and *Glyma18g03090*) around QTNs for salt tolerance index traits, one candidate GEI (*Glyma16g22630*) around iQEIs, and one candidate GEI (*Glyma02g38910*) around QEI for salt tolerance related traits were higher in landrace soybean than in bred soybean (Table 4). For example, the elite haplotype frequency of *Glyma07g18150* was 100%, 68.18%, and 56.73%, respectively, in wild, landrace, and improved soybeans, respectively (Fig. 3J). The results indicated that these candidate genes and GEIs had undergone domestication and improvement processes. In addition, the elite haplotypes in Table 4 can be used in soybean breeding for salt tolerance-related traits.

### The GEIs for salt tolerance related traits may respond to other environment stresses

When seeds are harvested in different environments, the seed formation process is influenced by their environment conditions in different environments. Although the experimental conditions for the salt tolerance experiments in this study are the same, the seeds themselves contain environmental influences, such as seed composition [69] and fatty acids [70, 71]. The different seed compositions have been shown to influence the tolerance [72–74]. In this study, 59 iQEIs were identified using multi-environment joint analysis, and two known genes and three candidate GEIs for salt tolerance index traits were identified. For example, the expression of the known gene *GmSK1* was simultaneously induced by several hormones and abiotic stresses, including abscisic acid (ABA), jasmonic acid (JA), salicylic acid (SA), NaCl, low temperature, and drought [54]. The candidate gene *Glyma04g41701* was homologous to *Arabidopsis AtWRKY30*, which is associated with oxidative and salinity tolerance during seed germination [75]. The results indicated that these genes and GEIs in this study may respond to multiple environmental stresses.

### Comparison between iQEIs for the four index-related traits and QEIs for the four salt stress-related traits between control and salt treatments

The iQEI and QEI detection is aimed at identifying the gene-by-environment interactions of salt tolerance related traits. In this study, we found the differences and similarities in the two types of results. First, seven QEIs were found to be within the same linkage disequilibrium intervals by the two types of analyses, such as snp57845 and snp57848, indicating the similarities (Tables S6 and S7). Then, most of the QEIs identified by the two types of analyses are different, possibly because their phenotypic values are different (salt tolerance indices and trait observations). In fact, the two types of analyses are complement each other to identify GEIs more comprehensively, as different GWAS methods described in Zhang et al. [76, 77].

Although this study used the phenotype datasets of Zhang et al. [32], who performed epistatic association mapping for salt tolerance using 135 SSR markers, the current results are more comprehensive and diverse. First, we used the new method (3VmrMLM) to associate richer markers (740,754 SNPs) with more phenotype datasets to identify more QTNs and QEIs for salt tolerance-related traits. Then, we identified candidate genes through comparative genomic analysis, gene differential expression analysis, KEGG pathway analysis, soybean gene annotation, SNP variation, haplotype analysis, and qRT-PCR experiment. Finally, we identified the elite haplotypes of genes that can be used in soybean breeding.

### The threshold of significant and suggested QTNs and QEIs

In our GWAS methodologies [34, 77], the *P*-value threshold for significant QTNs and QEIs is determined by the Bonferroni correction probability. As we know, this criterion is too strict and some important genes or GEIs might be missed [77]. To address this issue, suggested loci with the threshold of LOD score = 3.0 were considered in our previous methodological articles [34, 77]. If strong evidence supports the genes/GEIs around suggested QTNs/QEIs, these loci are valuable. In this study, six known salt tolerance-related genes, such as *GmNHX1*, and six candidate genes with strong evidence (differential expression analysis, gene annotation, *Arabidopsis* homologous genes, and haplotype analysis) were found to be around the suggested QTNs (Tables 1 and 3). More importantly, the 3VmrMLM method was proven to strictly control the false positive rate at the threshold of LOD score = 3.0 [34]. This approach has been widely adopted in the application studies of our GWAS methods [76–78].

## Conclusion

Around 258 QTNs and 59 iQEIs identified for four salt tolerance index related traits, 8 and 2 known salt tolerance genes were verified in previous studies, and 6 candidate genes and 3 candidate GEIs were predicted to be associated with these traits using multi-omics and bioinformatics analysis. Around 166 QEIs identified for four salt-tolerance-related traits between control and salt treatments, 6 salt-tolerance genes were verified in previous studies, and 5 candidate GEIs were predicted to be associated with salt stress, at least by haplotype analysis. In addition, the elite molecular modules of seven candidate genes with selection signs were extracted from wild soybean and could be applied to soybean molecular breeding. More importantly, the candidate gene *Glyma07g18150* was confirmed by qRT-PCR and predicted to play important roles in salt stress response*.* This study will provide important information for the genetic basis and breeding of salt tolerance in soybean.

### Supplementary Information

**Additional file 1: Figure S1. **Simple correlation analysis for salt tolerance index traits in 286 soybean accessions.** Figure S2. **Manhattan plots for salt tolerance index traits in soybean using 3VmrMLM.** Figure S3. **Relative expression levels of *Glyma06G04840* and *Glyma07G18150* under control and 6 h salt treatments.

**Additional file 2: Table S1.**Primer sequences used for qRT-PCR.** Table S2.**The summary of phenotypes and BLUP value of salt tolerance index traits in 286 soybeans accessions.** Table S3.**Two-way (genotypes and environments) ANOVA for soybean salt tolerance index traits.** Table S4.**QTNs for soybean salt tolerance index traits in a single environment using 3VmrMLM.** Table S5.**QTNs for soybean salt tolerance index traits in all the environments using 3VmrMLM.** Table S6.**QEIs for soybean salt tolerance index traits in all the environments using 3VmrMLM.** Table S7.**QEIs for soybean salt tolerance related traits between control and salt treatments using 3VmrMLM.** Table S8.**The KEGG pathway of genes in co-expression modules using KOBAS software.** Table S9. **The ANOVA for soybean salt tolerance index traits among different evolution types.

## Data Availability

The datasets that support the findings in this study are available in the Supplementary Material of this manuscript.
