# Supplementary material for: Identification of QTNs, QTN-by-environment interactions, and their candidate genes for salt tolerance related traits in soybean
Source: BMC Plant Biol. 2024 Apr 23;24:316. doi: 10.1186/s12870-024-05021-8 (PMC11036579; doi:10.1186/s12870-024-05021-8)
Supplement: Supplementary file 1 — Additional file 1: Figure S1. Simple correlation analysis for salt tolerance index traits in 286 soybean accessions. Figure S2. Manhattan plots for salt tolerance index traits in soybean using 3VmrMLM. Figure S3. Relative expression levels of Glyma06G04840 and Glyma07G18150 under control and 6 h salt treatments. [file 12870_2024_5021_MOESM1_ESM.docx]

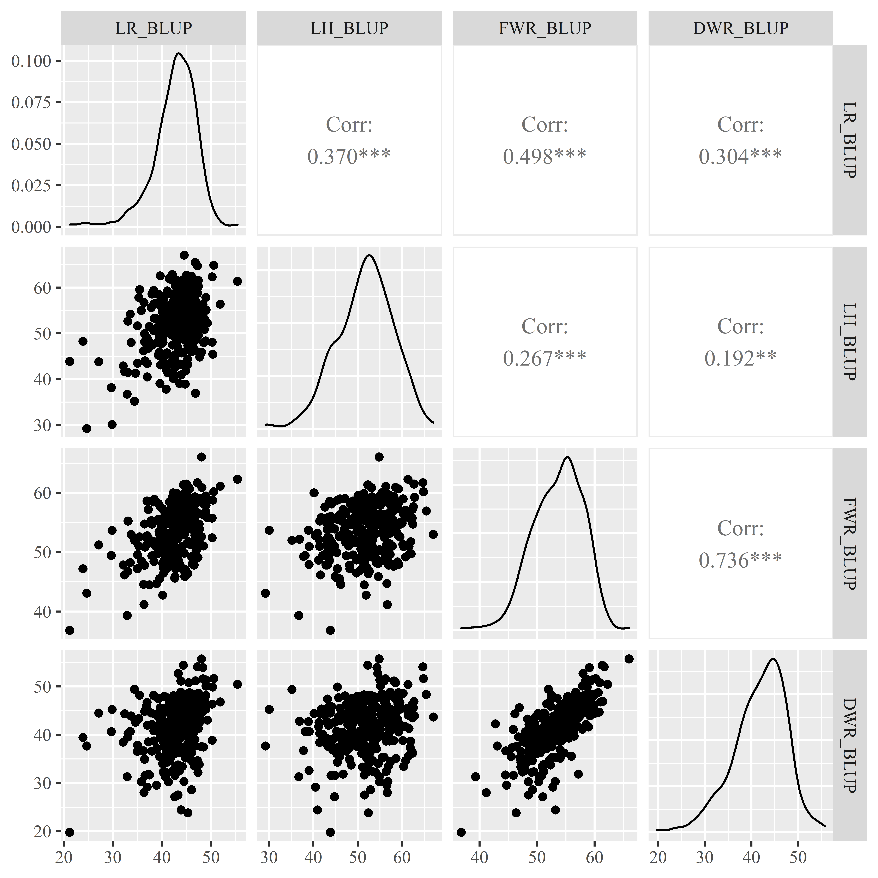


**Figure S1.** **Simple correlation analysis for salt tolerance index traits in 286 soybean accessions.** The upper triangle lists correlation coefficient and its significance. The lower triangle is the scatter of BLUP value. The diagonal is density map of BLUP values. ** and ***: significance at the 0.01 and 0.001 probability levels, respectively.


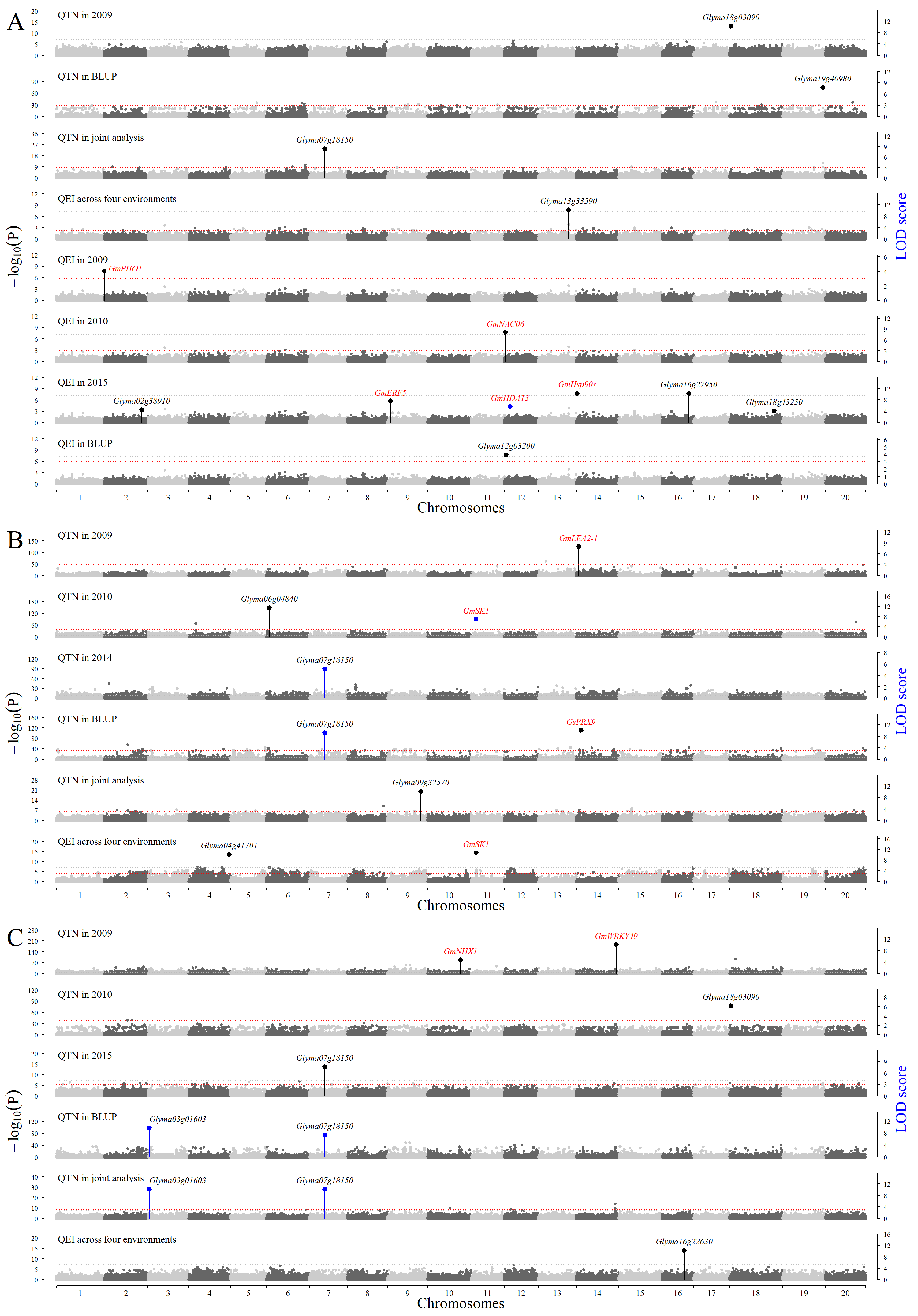


**Figure S2.** Manhattan plots for salt tolerance index traits in soybean using 3VmrMLM. A: the length of hypocotyls; B: the dry weights of roots; C: the fresh weights of roots. The small figures of QTN in 2009, 2010, 2014, 2015 and BLUP were QTNs, detected using single environment module in software IIIVmrMLM. QTN in joint analysis and QEIs across four environments were QTNs and QTN-by-environment interactions (QEIs), respectively, detected using multi-environment joint analysis module in software IIIVmrMLM. QEI in 2009, 2010, 2014, 2015, and BLUP were the QEI between control and salt stress, detected using multi-environment joint analysis module in software IIIVmrMLM. The black (one) and blue (multiple) lines indicate the number of times that the QTN/QEI was identified. Known genes, candidate genes, and gene-by-environment interactions (GEIs) were marked with red, black, and magenta colors, respectively.


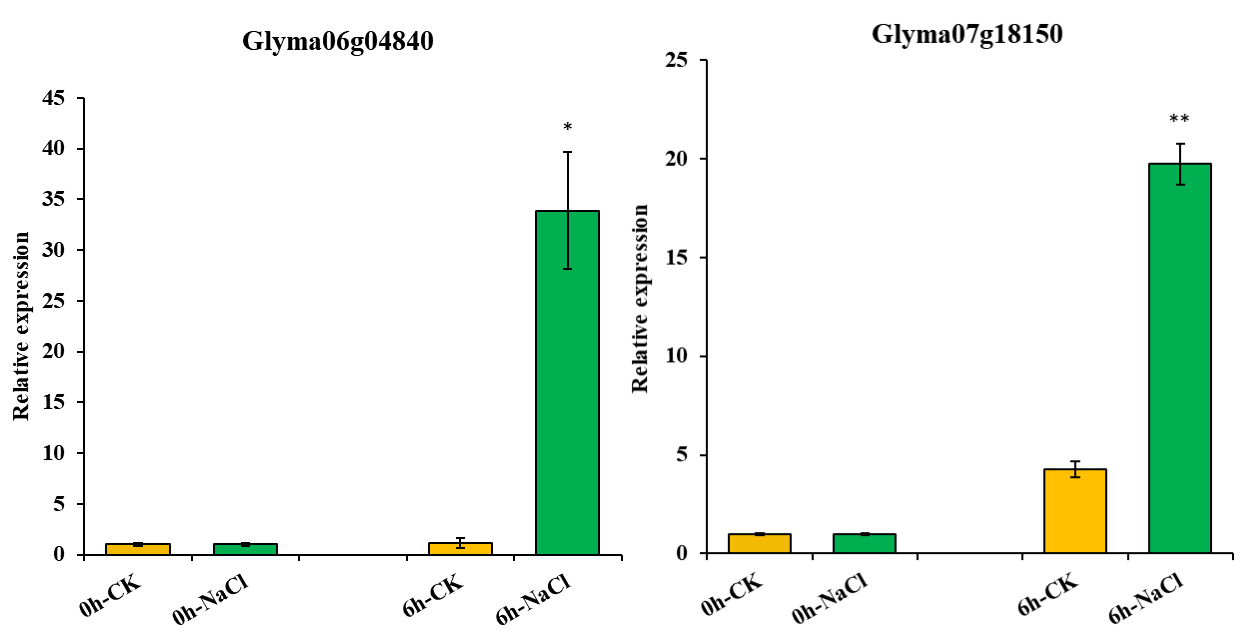


**Figure S3. Relative expression levels of *Glyma06G04840* and *Glyma07G18150* under control and 6 h salt treatments. * and **: the 0.05 and 0.01 probability levels of significance, respectively.**
