# Supplementary material for: Identification of QTNs, QTN-by-environment interactions, and their candidate genes for salt tolerance related traits in soybean
Source: BMC Plant Biol. 2024 Apr 23;24:316. doi: 10.1186/s12870-024-05021-8 (PMC11036579; doi:10.1186/s12870-024-05021-8)
Supplement: Supplementary file 2 — Additional file 2: Table S1.Primer sequences used for qRT-PCR. Table S2.The summary of phenotypes and BLUP value of salt tolerance index traits in 286 soybeans accessions. Table S3.Two-way (genotypes and environments) ANOVA for soybean salt tolerance index traits. Table S4.QTNs for soybean salt tolerance index traits in a single environment using 3VmrMLM. Table S5.QTNs for soybean salt tolerance index traits in all the environments using 3VmrMLM. Table S6.QEIs for soybean salt tolerance index traits in all the environments using 3VmrMLM. Table S7.QEIs for soybean salt tolerance related traits between control and salt treatments using 3VmrMLM. Table S8.The KEGG pathway of genes in co-expression modules using KOBAS software. Table S9. The ANOVA for soybean salt tolerance index traits among different evolution types. [file 12870_2024_5021_MOESM2_ESM.docx]

**Table S1. Primer sequences used for qRT-PCR**

| **Primer name** | **Sequences (5′→3′)** |
| --- | --- |
| Glyma06G04840-F | AACAATTCTGCGCCGCTATC |
| Glyma06G04840-R | CAAGGCCTGTCCTGTCGAA |
| Glyma07G18150-F | TGGCGAGGATGGTTCAGAAG |
| Glyma07G18150-R | GCTGCATTGCCAACTATCGG |
| Actin11-F | CGGTGGTTCTATCTTGGCATC |
| Actin11-R | GTCTTTCGCTTCAATAACCCTA |

**Table S2. The summary of phenotypes and BLUP value of salt tolerance index traits in 286 soybeans accessions.**

| **Trait** | **Env** | **Mean** | **SD** | **CV** | **Min** | **Max** | **Range** | **Skew** | **Kurt** | **Heritability** |
| --- | --- | --- | --- | --- | --- | --- | --- | --- | --- | --- |
| DWR | 2009 | 51.65 | 15.89 | 30.75 | 0.00 | 87.37 | 87.37 | -0.55 | 0.09 | 47.46 |
|  | 2010 | 33.04 | 20.95 | 63.40 | -108.33 | 71.43 | 179.76 | -1.69 | 7.58 |  |
|  | 2014 | 45.87 | 20.73 | 45.19 | -34.62 | 100.00 | 134.62 | -1.06 | 1.69 |  |
|  | 2015 | 36.31 | 20.32 | 55.95 | -50.00 | 77.50 | 127.50 | -0.74 | 1.02 |  |
|  | BLUP | 41.80 | 5.69 | 13.62 | 19.77 | 55.71 | 35.93 | -0.65 | 0.70 |  |
| FWR | 2009 | 63.52 | 14.03 | 22.09 | 11.11 | 95.86 | 84.75 | -0.89 | 1.26 | 50.85 |
|  | 2010 | 50.69 | 12.74 | 25.13 | 0.31 | 82.69 | 82.38 | -0.39 | 0.75 |  |
|  | 2014 | 52.27 | 12.73 | 24.35 | -6.16 | 85.67 | 91.83 | -0.93 | 2.32 |  |
|  | 2015 | 47.85 | 15.02 | 31.38 | -11.33 | 80.54 | 91.87 | -1.00 | 1.79 |  |
|  | BLUP | 53.62 | 4.35 | 8.12 | 36.80 | 66.00 | 29.20 | -0.47 | 0.39 |  |
| LH | 2009 | 50.62 | 14.67 | 28.97 | -0.26 | 82.57 | 82.83 | -0.44 | 0.03 | 64.74 |
|  | 2010 | 46.57 | 15.43 | 33.14 | 5.47 | 79.27 | 73.80 | -0.30 | -0.34 |  |
|  | 2014 | 59.23 | 11.42 | 19.28 | 9.95 | 81.71 | 71.76 | -1.27 | 3.23 |  |
|  | 2015 | 50.20 | 14.92 | 29.71 | -9.76 | 79.14 | 88.90 | -0.59 | 0.66 |  |
|  | BLUP | 51.66 | 6.33 | 12.26 | 29.20 | 67.07 | 37.87 | -0.39 | 0.24 |  |
| LR | 2009 | 42.91 | 13.99 | 32.60 | -25.37 | 68.90 | 94.27 | -1.07 | 1.79 | 55.19 |
|  | 2010 | 44.90 | 10.14 | 22.57 | 1.62 | 70.23 | 68.61 | -0.91 | 2.34 |  |
|  | 2014 | 40.51 | 13.68 | 33.76 | -26.67 | 70.47 | 97.14 | -1.34 | 3.85 |  |
|  | 2015 | 42.43 | 12.38 | 29.19 | -12.14 | 76.49 | 88.63 | -0.95 | 1.57 |  |
|  | BLUP | 42.65 | 4.50 | 10.54 | 21.22 | 55.40 | 34.18 | -1.23 | 3.32 |  |

DWR: the dry weights of roots; FWR: the fresh weights of roots; LH: the length of hypocotyls; LR: the length of main root; SD: standard deviation; Min: minimum; Max: maximum; CV: coefficient of variation; Skew: skewness; Kurt: kurtosis.

**Table S3. Two-way (genotypes and environments) ANOVA for soybean salt tolerance index traits**

| **Trait** | **Genotype** | | |  | **Environment** | | |
| --- | --- | --- | --- | --- | --- | --- | --- |
|  | **DF** | ***F* value** | ***P* value** |  | **DF** | ***F* value** | ***P* value** |
| LR | 285 | 2.18 | 1.81E-17 |  | 3 | 6.98 | 1.22E-04 |
| LH | 285 | 2.76 | 8.57E-29 |  | 3 | 55.30 | 1.77E-32 |
| FWR | 285 | 1.98 | 1.10E-13 |  | 3 | 87.09 | 1.01E-48 |
| DWR | 285 | 1.84 | 3.62E-11 |  | 3 | 65.25 | 1.10E-37 |

DWR: the dry weights of roots; FWR: the fresh weights of roots; LH: the length of hypocotyls; LR: the length of main root; DF: degree of freedom

**Table S4. QTNs for soybean salt tolerance index traits in a single environment using 3VmrMLM.**

| **Trait** | **Env** | **Marker** | **Chr** | **Pos (bp)** | **LOD** | **Add** | **Dom** | **Var** | **r^2^(%)** | ***P*-value** | **Significance** |
| --- | --- | --- | --- | --- | --- | --- | --- | --- | --- | --- | --- |
| LR | 2009 | Gm05:26135344 | 5 | 26135344 | 3.28 | 0.77 | -10.71 | 5.71 | 2.92 | 5.27E-04 | SUG |
| LR | 2009 | snp24293 | 5 | 32472416 | 7.07 | 1.38 | -38.08 | 11.79 | 6.03 | 8.51E-08 | SUG |
| LR | 2009 | Gm06:34098367 | 6 | 34098367 | 58.85 | 13.74 | 3.83 | 9.58 | 4.89 | 1.42E-59 | SIG |
| LR | 2009 | Gm08:43340217 | 8 | 43340217 | 10.96 | 4.55 |  | 6.31 | 3.23 | 1.21E-12 | SIG |
| LR | 2009 | snp46379 | 9 | 38423871 | 8.47 | -3.97 | -2.63 | 4.01 | 2.05 | 3.39E-09 | SIG |
| LR | 2009 | snp46783 | 9 | 41240076 | 5.03 | 2.99 | -3.34 | 5.16 | 2.64 | 9.28E-06 | SUG |
| LR | 2009 | Gm17:22873804 | 17 | 22873804 | 4.39 | 0.15 | -17.13 | 7.71 | 3.94 | 4.07E-05 | SUG |
| LR | 2009 | Gm19:10294352 | 19 | 10294352 | 18.51 | 6.11 |  | 5.97 | 3.05 | 2.63E-20 | SIG |
| LR | 2009 | Gm20:38693249 | 20 | 38693249 | 3.68 | -1.37 | 6.53 | 6.31 | 3.22 | 2.07E-04 | SUG |
| LR | 2010 | snp11076 | 3 | 1565191 | 13.58 | -2.67 | -39.48 | 6.68 | 6.50 | 2.66E-14 | SIG |
| LR | 2010 | Gm07:22311500 | 7 | 22311500 | 13.49 | -3.56 | 3.70 | 4.11 | 4.00 | 3.22E-14 | SIG |
| LR | 2010 | snp35525 | 7 | 36011307 | 3.99 | -0.14 | 21.62 | 3.48 | 3.39 | 1.02E-04 | SUG |
| LR | 2010 | snp37756 | 8 | 7535754 | 8.92 | -1.62 | -17.29 | 4.95 | 4.82 | 1.21E-09 | SIG |
| LR | 2010 | Gm11:6545943 | 11 | 6545943 | 4.25 | 0.31 | -6.26 | 3.61 | 3.52 | 5.69E-05 | SUG |
| LR | 2010 | snp54902 | 11 | 18083326 | 10.99 | 3.16 | -5.07 | 4.23 | 4.12 | 1.02E-11 | SIG |
| LR | 2010 | snp69518 | 14 | 16722480 | 8.45 | 2.64 | -7.29 | 1.53 | 1.49 | 3.59E-09 | SIG |
| LR | 2010 | Gm16:9986883 | 16 | 9986883 | 80.61 | 12.59 | 5.24 | 6.91 | 6.72 | 2.48E-81 | SIG |
| LR | 2010 | Gm17:32131195 | 17 | 32131195 | 41.30 | 7.11 |  | 8.58 | 8.35 | 2.89E-43 | SIG |
| LR | 2014 | Gm01:4555090 | 1 | 4555090 | 8.41 | 0.38 | -18.54 | 11.45 | 6.12 | 3.93E-09 | SIG |
| LR | 2014 | Gm01:28178629 | 1 | 28178629 | 5.46 | -0.18 | -11.47 | 6.65 | 3.56 | 3.50E-06 | SUG |
| LR | 2014 | Gm02:22510359 | 2 | 22510359 | 17.99 | -4.00 | -19.60 | 7.21 | 3.86 | 1.03E-18 | SIG |
| LR | 2014 | snp25655 | 6 | 1578396 | 23.70 | 6.10 |  | 6.77 | 3.62 | 1.50E-25 | SIG |
| LR | 2014 | Gm06:44259955 | 6 | 44259955 | 33.69 | -7.53 | -8.14 | 10.47 | 5.60 | 2.05E-34 | SIG |
| LR | 2014 | Gm12:17491076 | 12 | 17491076 | 64.30 | 12.35 | -2.89 | 8.78 | 4.70 | 5.08E-65 | SIG |
| LR | 2014 | snp67772 | 14 | 2907230 | 6.58 | 0.17 | -14.79 | 8.64 | 4.62 | 2.63E-07 | SUG |
| LR | 2014 | snp80696 | 16 | 16337878 | 17.74 | 4.89 | 9.94 | 10.56 | 5.64 | 1.82E-18 | SIG |
| LR | 2014 | snp94210 | 18 | 56231434 | 7.38 | -1.73 | -16.60 | 6.79 | 3.63 | 4.16E-08 | SIG |
| LR | 2014 | Gm20:27434549 | 20 | 27434549 | 6.49 | -0.63 | -6.04 | 5.27 | 2.82 | 3.21E-07 | SUG |
| LR | 2015 | Gm03:40724131 | 3 | 40724131 | 10.35 | 2.96 |  | 2.22 | 1.45 | 5.02E-12 | SIG |
| LR | 2015 | snp16025 | 4 | 1512355 | 4.70 | 2.01 | -1.02 | 3.75 | 2.44 | 1.98E-05 | SUG |
| LR | 2015 | Gm05:5939611 | 5 | 5939611 | 9.78 | -1.58 | -10.80 | 4.15 | 2.70 | 1.64E-10 | SIG |
| LR | 2015 | snp25289 | 5 | 40410433 | 17.72 | 4.07 | 0.93 | 9.90 | 6.45 | 1.91E-18 | SIG |
| LR | 2015 | Gm06:38426950 | 6 | 38426950 | 10.94 | -3.25 | 1.87 | 4.07 | 2.65 | 1.16E-11 | SIG |
| LR | 2015 | snp36597 | 7 | 43811572 | 40.70 | 6.60 | -15.25 | 6.39 | 4.17 | 2.00E-41 | SIG |
| LR | 2015 | Gm10:11312903 | 10 | 11312903 | 14.07 | 3.50 |  | 5.39 | 3.51 | 8.23E-16 | SIG |
| LR | 2015 | Gm13:29091138 | 13 | 29091138 | 9.60 | 1.45 | -13.66 | 7.19 | 4.69 | 2.54E-10 | SIG |
| LR | 2015 | snp72395 | 14 | 48681142 | 5.10 | 0.66 | 22.66 | 3.53 | 2.30 | 8.02E-06 | SUG |
| LR | 2015 | Gm15:14407770 | 15 | 14407770 | 8.90 | 3.22 | 0.90 | 4.84 | 3.16 | 1.27E-09 | SIG |
| LR | 2015 | snp80040 | 16 | 7789725 | 6.89 | 2.42 | 1.80 | 4.84 | 3.16 | 1.29E-07 | SUG |
| LR | 2015 | snp81355 | 16 | 24076877 | 4.98 | -1.95 | -2.67 | 3.88 | 2.53 | 1.04E-05 | SUG |
| LR | 2015 | Gm18:14065740 | 18 | 14065740 | 7.16 | 2.43 |  | 4.12 | 2.69 | 9.25E-09 | SIG |
| LR | 2015 | Gm18:30056777 | 18 | 30056777 | 19.69 | -4.64 | 1.58 | 7.34 | 4.78 | 2.05E-20 | SIG |
| LR | 2015 | snp102171 | 20 | 14100232 | 37.01 | 5.77 | -15.78 | 13.36 | 8.71 | 9.87E-38 | SIG |
| LR | BLUP | snp16966 | 4 | 7659998 | 4.34 | 0.09 | 7.84 | 0.62 | 3.09 | 4.62E-05 | SUG |
| LR | BLUP | snp24293 | 5 | 32472416 | 9.22 | -0.97 | -8.60 | 0.49 | 2.40 | 5.99E-10 | SIG |
| LR | BLUP | Gm06:44259955 | 6 | 44259955 | 21.03 | -1.99 | -1.18 | 0.72 | 3.59 | 9.35E-22 | SIG |
| LR | BLUP | Gm07:30087591 | 7 | 30087591 | 9.98 | 1.26 |  | 0.64 | 3.18 | 1.21E-11 | SIG |
| LR | BLUP | snp68147 | 14 | 4917939 | 22.43 | 2.38 | 0.86 | 0.94 | 4.65 | 3.76E-23 | SIG |
| LR | BLUP | Gm17:32131195 | 17 | 32131195 | 27.47 | 2.24 |  | 0.87 | 4.32 | 2.41E-29 | SIG |
| LR | BLUP | snp99798 | 19 | 43776160 | 21.28 | 1.93 | 1.66 | 0.60 | 2.96 | 5.20E-22 | SIG |
| LR | BLUP | snp102171 | 20 | 14100232 | 10.23 | 0.58 | -6.79 | 1.48 | 7.32 | 5.83E-11 | SIG |
| LH | 2009 | Gm08:10929076 | 8 | 10929076 | 85.69 | -18.30 | -36.07 | 16.75 | 7.79 | 2.04E-86 | SIG |
| LH | 2009 | Gm09:26948106 | 9 | 26948106 | 3.35 | -0.37 | -10.56 | 5.58 | 2.60 | 4.45E-04 | SUG |
| LH | 2009 | Gm10:31420578 | 10 | 31420578 | 3.05 | 0.06 | 10.92 | 5.52 | 2.57 | 8.83E-04 | SUG |
| LH | 2009 | snp55389 | 11 | 25481063 | 22.15 | 6.87 | -16.55 | 9.53 | 4.43 | 7.14E-23 | SIG |
| LH | 2009 | snp56491 | 11 | 36449725 | 4.25 | -2.95 | 1.02 | 7.36 | 3.42 | 5.69E-05 | SUG |
| LH | 2009 | snp61804 | 13 | 3257156 | 10.91 | -3.13 | -26.08 | 12.94 | 6.02 | 1.23E-11 | SIG |
| LH | 2009 | snp83317 | 16 | 35364110 | 6.05 | 3.55 | 1.16 | 11.02 | 5.12 | 8.88E-07 | SUG |
| LH | 2009 | Gm17:30702653 | 17 | 30702653 | 27.81 | -6.06 | -17.97 | 14.11 | 6.56 | 1.54E-28 | SIG |
| LH | 2009 | Gm18:2022673 | 18 | 2022673 | 22.13 | 4.12 | 9.70 | 12.42 | 5.77 | 7.46E-23 | SIG |
| LH | 2010 | snp14825 | 3 | 39715844 | 22.13 | 7.67 | 0.03 | 17.16 | 7.03 | 7.34E-23 | SIG |
| LH | 2010 | snp15542 | 3 | 45284400 | 13.85 | 5.82 | -2.62 | 8.42 | 3.45 | 1.40E-14 | SIG |
| LH | 2010 | Gm05:2390530 | 5 | 2390530 | 10.41 | 4.68 | -6.83 | 12.01 | 4.92 | 3.92E-11 | SIG |
| LH | 2010 | snp26880 | 6 | 10020149 | 21.88 | 7.53 | 5.70 | 16.26 | 6.66 | 1.31E-22 | SIG |
| LH | 2010 | Gm09:37505213 | 9 | 37505213 | 13.30 | 5.64 |  | 11.45 | 4.69 | 5.02E-15 | SIG |
| LH | 2010 | snp65440 | 13 | 32037766 | 34.48 | 10.04 | 7.38 | 13.06 | 5.35 | 3.28E-35 | SIG |
| LH | 2010 | snp67505 | 14 | 1073440 | 15.91 | 6.26 | -3.94 | 8.71 | 3.57 | 1.23E-16 | SIG |
| LH | 2010 | snp75710 | 15 | 21180259 | 9.79 | 4.57 | -8.69 | 17.71 | 7.26 | 1.64E-10 | SIG |
| LH | 2010 | snp77361 | 15 | 42317139 | 12.08 | 5.40 | 0.73 | 9.03 | 3.70 | 8.36E-13 | SIG |
| LH | 2010 | snp84610 | 17 | 7498627 | 5.71 | 4.21 | -0.08 | 5.35 | 2.19 | 1.97E-06 | SUG |
| LH | 2014 | snp10954 | 3 | 744345 | 15.07 | 4.28 | -4.32 | 3.81 | 2.92 | 8.52E-16 | SIG |
| LH | 2014 | snp15542 | 3 | 45284400 | 30.21 | 6.36 | 10.09 | 9.42 | 7.22 | 6.11E-31 | SIG |
| LH | 2014 | snp20282 | 4 | 43742331 | 31.25 | 6.62 |  | 3.77 | 2.89 | 3.70E-33 | SIG |
| LH | 2014 | snp37188 | 8 | 3498881 | 22.87 | -5.48 | 2.70 | 6.30 | 4.83 | 1.36E-23 | SIG |
| LH | 2014 | snp42553 | 8 | 46652013 | 5.14 | 1.22 | -15.26 | 5.14 | 3.94 | 7.30E-06 | SUG |
| LH | 2014 | snp42647 | 9 | 336033 | 24.79 | 5.74 | 3.15 | 3.79 | 2.91 | 1.64E-25 | SIG |
| LH | 2014 | Gm09:25751703 | 9 | 25751703 | 8.60 | -1.78 | -19.19 | 5.61 | 4.30 | 2.50E-09 | SIG |
| LH | 2014 | snp93895 | 18 | 55088428 | 10.06 | -3.35 | 6.47 | 3.90 | 2.99 | 8.71E-11 | SIG |
| LH | 2015 | snp6882 | 2 | 10437962 | 9.80 | 2.91 | 2.78 | 5.13 | 2.30 | 1.57E-10 | SIG |
| LH | 2015 | snp7033 | 2 | 11386741 | 15.23 | 3.84 | 0.41 | 12.05 | 5.42 | 5.93E-16 | SIG |
| LH | 2015 | Gm02:40275338 | 2 | 40275338 | 10.25 | 3.14 | -1.60 | 5.99 | 2.69 | 5.62E-11 | SIG |
| LH | 2015 | Gm03:13136647 | 3 | 13136647 | 9.73 | 2.83 | -3.57 | 3.07 | 1.38 | 1.87E-10 | SIG |
| LH | 2015 | snp25531 | 6 | 594638 | 3.74 | 1.55 | -3.54 | 3.01 | 1.35 | 1.82E-04 | SUG |
| LH | 2015 | snp30182 | 6 | 42784786 | 7.08 | 2.50 | -0.43 | 4.54 | 2.04 | 8.34E-08 | SUG |
| LH | 2015 | snp30820 | 6 | 46904335 | 17.37 | -3.35 | -18.50 | 3.41 | 1.53 | 4.23E-18 | SIG |
| LH | 2015 | snp35490 | 7 | 35814041 | 3.43 | 1.71 | -1.24 | 2.79 | 1.26 | 3.69E-04 | SUG |
| LH | 2015 | Gm08:19476084 | 8 | 19476084 | 60.77 | 7.63 | 13.62 | 6.55 | 2.94 | 1.71E-61 | SIG |
| LH | 2015 | Gm10:10609119 | 10 | 10609119 | 10.49 | 3.01 |  | 3.78 | 1.70 | 3.67E-12 | SIG |
| LH | 2015 | snp57277 | 12 | 2519150 | 8.77 | -0.31 | 9.27 | 7.02 | 3.16 | 1.71E-09 | SIG |
| LH | 2015 | snp59404 | 12 | 17545640 | 8.38 | -0.44 | -15.56 | 6.55 | 2.94 | 4.13E-09 | SIG |
| LH | 2015 | snp59905 | 12 | 25510360 | 7.87 | -3.82 | 0.18 | 4.32 | 1.94 | 1.35E-08 | SIG |
| LH | 2015 | Gm13:8915449 | 13 | 8915449 | 16.74 | 3.86 | -4.52 | 5.06 | 2.28 | 1.80E-17 | SIG |
| LH | 2015 | snp64572 | 13 | 27798265 | 9.46 | -2.90 | 0.06 | 5.63 | 2.53 | 3.48E-10 | SIG |
| LH | 2015 | Gm13:44196413 | 13 | 44196413 | 4.60 | 1.00 | -6.65 | 3.58 | 1.61 | 2.49E-05 | SUG |
| LH | 2015 | snp74860 | 15 | 15027624 | 5.76 | 2.27 | 0.88 | 4.75 | 2.13 | 1.75E-06 | SUG |
| LH | 2015 | Gm15:43429044 | 15 | 43429044 | 6.03 | -2.63 | -1.30 | 4.75 | 2.14 | 9.33E-07 | SUG |
| LH | 2015 | snp91316 | 18 | 27386631 | 16.48 | 0.49 | 4.73 | 4.64 | 2.09 | 3.29E-17 | SIG |
| LH | 2015 | snp94728 | 18 | 58872727 | 23.08 | 4.76 | -0.90 | 6.73 | 3.02 | 8.27E-24 | SIG |
| LH | 2015 | Gm19:24683941 | 19 | 24683941 | 24.17 | 5.11 | 2.60 | 4.09 | 1.84 | 6.73E-25 | SIG |
| LH | 2015 | snp98076 | 19 | 31181048 | 4.26 | -10.70 | -0.47 | 3.29 | 1.48 | 5.51E-05 | SUG |
| LH | BLUP | snp15542 | 3 | 45284400 | 19.67 | 2.27 | -1.80 | 1.43 | 3.56 | 2.14E-20 | SIG |
| LH | BLUP | snp24367 | 5 | 33073935 | 8.26 | 1.30 | -3.03 | 1.13 | 2.82 | 5.45E-09 | SIG |
| LH | BLUP | snp26886 | 6 | 10049699 | 13.49 | 1.76 | 4.24 | 0.91 | 2.27 | 3.28E-14 | SIG |
| LH | BLUP | snp30631 | 6 | 45611572 | 12.18 | -1.76 | -0.67 | 2.93 | 7.31 | 6.67E-13 | SIG |
| LH | BLUP | snp55389 | 11 | 25481063 | 17.79 | -1.82 | -9.50 | 0.91 | 2.27 | 1.62E-18 | SIG |
| LH | BLUP | snp67505 | 14 | 1073440 | 14.72 | 1.90 | 2.69 | 0.73 | 1.82 | 1.90E-15 | SIG |
| LH | BLUP | Gm15:6725523 | 15 | 6725523 | 39.34 | 2.73 | 5.11 | 1.31 | 3.25 | 4.55E-40 | SIG |
| LH | BLUP | snp74927 | 15 | 15393585 | 5.08 | -0.20 | 4.97 | 1.15 | 2.87 | 8.40E-06 | SUG |
| LH | BLUP | Gm15:20375281 | 15 | 20375281 | 11.09 | -0.98 | -6.46 | 1.23 | 3.06 | 8.11E-12 | SIG |
| LH | BLUP | Gm19:8398798 | 19 | 8398798 | 7.20 | -0.85 | 6.81 | 1.24 | 3.09 | 6.34E-08 | SIG |
| LH | BLUP | snp98807 | 19 | 37061537 | 15.44 | 1.97 | 0.94 | 0.92 | 2.30 | 3.68E-16 | SIG |
| LH | BLUP | snp100307 | 19 | 47249031 | 7.76 | 1.34 | -1.51 | 1.82 | 4.53 | 1.76E-08 | SIG |
| LH | BLUP | snp102516 | 20 | 18936372 | 18.86 | 3.08 | 1.68 | 1.19 | 2.97 | 1.37E-19 | SIG |
| FWR | 2009 | snp7676 | 2 | 17825596 | 8.71 | -1.96 | -38.09 | 5.11 | 2.60 | 1.96E-09 | SIG |
| FWR | 2009 | snp15697 | 3 | 46612389 | 12.59 | -3.60 | 5.28 | 13.10 | 6.65 | 2.58E-13 | SIG |
| FWR | 2009 | snp16602 | 4 | 5487907 | 9.23 | 3.17 | -2.82 | 5.08 | 2.58 | 5.85E-10 | SIG |
| FWR | 2009 | Gm04:36500408 | 4 | 36500408 | 21.78 | 4.98 | -8.00 | 6.81 | 3.46 | 1.66E-22 | SIG |
| FWR | 2009 | snp20160 | 4 | 43093092 | 3.24 | 1.07 | 6.72 | 2.16 | 1.10 | 5.75E-04 | SUG |
| FWR | 2009 | Gm06:31270040 | 6 | 31270040 | 7.45 | 0.75 | -8.82 | 7.85 | 3.99 | 3.56E-08 | SIG |
| FWR | 2009 | snp39068 | 8 | 15847656 | 11.43 | 1.14 | -17.25 | 12.53 | 6.36 | 3.76E-12 | SIG |
| FWR | 2009 | snp41926 | 8 | 42795977 | 20.28 | 4.92 | -0.05 | 10.08 | 5.12 | 5.25E-21 | SIG |
| FWR | 2009 | Gm10:34118947 | 10 | 34118947 | 5.57 | -2.41 |  | 3.10 | 1.57 | 4.13E-07 | SUG |
| FWR | 2009 | snp50832 | 10 | 38837251 | 4.79 | -2.29 | 0.55 | 4.00 | 2.03 | 1.62E-05 | SUG |
| FWR | 2009 | snp51034 | 10 | 40177093 | 7.43 | -0.14 | -32.17 | 7.72 | 3.92 | 3.71E-08 | SIG |
| FWR | 2009 | Gm14:14432588 | 14 | 14432588 | 24.36 | -5.48 |  | 8.81 | 4.47 | 3.29E-26 | SIG |
| FWR | 2009 | snp70623 | 14 | 30110481 | 38.81 | 7.45 | -2.02 | 10.50 | 5.34 | 1.56E-39 | SIG |
| FWR | 2009 | Gm14:47141962 | 14 | 47141962 | 16.19 | 4.30 |  | 4.47 | 2.27 | 5.94E-18 | SIG |
| FWR | 2009 | Gm16:5515 | 16 | 5515 | 68.01 | 11.64 | 2.81 | 5.62 | 2.85 | 9.73E-69 | SIG |
| FWR | 2009 | snp89461 | 18 | 10548530 | 22.41 | 5.16 | 10.00 | 6.28 | 3.19 | 3.91E-23 | SIG |
| FWR | 2009 | snp92434 | 18 | 46392083 | 22.90 | -3.32 | -38.65 | 14.76 | 7.50 | 1.25E-23 | SIG |
| FWR | 2010 | snp18256 | 4 | 19256411 | 4.17 | -0.84 | -18.35 | 6.94 | 4.27 | 6.76E-05 | SUG |
| FWR | 2010 | Gm07:25021242 | 7 | 25021242 | 7.57 | 0.02 | -18.55 | 14.69 | 9.05 | 2.68E-08 | SIG |
| FWR | 2010 | snp65835 | 13 | 34490340 | 4.88 | -1.56 | -25.51 | 7.01 | 4.32 | 1.32E-05 | SUG |
| FWR | 2010 | snp88309 | 18 | 2040047 | 6.22 | 3.39 | -6.18 | 10.59 | 6.53 | 5.98E-07 | SUG |
| FWR | 2014 | snp45331 | 9 | 29736937 | 11.01 | -1.76 | -36.33 | 13.93 | 8.60 | 9.85E-12 | SIG |
| FWR | 2014 | Gm13:6788766 | 13 | 6788766 | 28.30 | -7.64 | -1.99 | 5.91 | 3.65 | 5.02E-29 | SIG |
| FWR | 2014 | Gm14:41084109 | 14 | 41084109 | 12.22 | 4.45 |  | 4.15 | 2.56 | 6.25E-14 | SIG |
| FWR | 2014 | snp74139 | 15 | 10918948 | 11.93 | -2.32 | -23.35 | 11.23 | 6.93 | 1.18E-12 | SIG |
| FWR | 2014 | snp76717 | 15 | 34078158 | 4.58 | -1.64 | 11.46 | 5.82 | 3.59 | 2.60E-05 | SUG |
| FWR | 2014 | Gm15:50358420 | 15 | 50358420 | 19.82 | -6.09 | 2.31 | 5.96 | 3.68 | 1.51E-20 | SIG |
| FWR | 2014 | snp87615 | 17 | 39421523 | 4.73 | -1.80 | 18.95 | 4.80 | 2.96 | 1.88E-05 | SUG |
| FWR | 2014 | Gm18:35621686 | 18 | 35621686 | 17.71 | 5.30 | -9.23 | 8.24 | 5.09 | 1.93E-18 | SIG |
| FWR | 2014 | Gm20:4797841 | 20 | 4797841 | 19.35 | 5.93 | 0.59 | 6.98 | 4.31 | 4.46E-20 | SIG |
| FWR | 2015 | Gm01:7467798 | 1 | 7467798 | 4.36 | -0.85 | -10.60 | 2.98 | 1.32 | 4.35E-05 | SUG |
| FWR | 2015 | Gm02:34582296 | 2 | 34582296 | 15.98 | -0.77 | -7.86 | 11.63 | 5.16 | 1.06E-16 | SIG |
| FWR | 2015 | snp18778 | 4 | 29123824 | 9.75 | 2.95 | 6.68 | 7.07 | 3.14 | 1.78E-10 | SIG |
| FWR | 2015 | Gm06:49646754 | 6 | 49646754 | 3.19 | -0.10 | 10.43 | 3.09 | 1.37 | 6.46E-04 | SUG |
| FWR | 2015 | snp31998 | 7 | 3954121 | 4.08 | -1.96 | -2.38 | 3.68 | 1.63 | 8.37E-05 | SUG |
| FWR | 2015 | Gm07:17751732 | 7 | 17751732 | 7.67 | 0.23 | -9.31 | 7.42 | 3.29 | 2.15E-08 | SIG |
| FWR | 2015 | snp40117 | 8 | 22428477 | 5.32 | -2.34 | 1.58 | 5.31 | 2.35 | 4.79E-06 | SUG |
| FWR | 2015 | snp46833 | 9 | 41501976 | 5.93 | 2.47 | 0.98 | 3.94 | 1.75 | 1.18E-06 | SUG |
| FWR | 2015 | snp48500 | 10 | 7178841 | 30.00 | 1.11 | 6.79 | 6.34 | 2.81 | 1.01E-30 | SIG |
| FWR | 2015 | Gm10:17870796 | 10 | 17870796 | 21.87 | -3.94 | -13.93 | 5.33 | 2.36 | 1.36E-22 | SIG |
| FWR | 2015 | Gm11:23564750 | 11 | 23564750 | 4.01 | 1.32 | -7.63 | 3.05 | 1.35 | 9.67E-05 | SUG |
| FWR | 2015 | Gm13:13489819 | 13 | 13489819 | 21.46 | -3.90 | -12.68 | 5.14 | 2.28 | 3.44E-22 | SIG |
| FWR | 2015 | Gm13:14770973 | 13 | 14770973 | 7.24 | -0.06 | 9.65 | 6.84 | 3.04 | 5.79E-08 | SIG |
| FWR | 2015 | Gm13:20216708 | 13 | 20216708 | 7.69 | 1.70 | -11.32 | 6.45 | 2.86 | 2.03E-08 | SIG |
| FWR | 2015 | snp67880 | 14 | 3426582 | 4.56 | 2.18 | 0.04 | 3.50 | 1.55 | 2.76E-05 | SUG |
| FWR | 2015 | snp71649 | 14 | 44645175 | 32.01 | 6.35 |  | 2.84 | 1.26 | 6.43E-34 | SIG |
| FWR | 2015 | Gm16:18475406 | 16 | 18475406 | 3.43 | 0.54 | -5.77 | 3.36 | 1.49 | 3.76E-04 | SUG |
| FWR | 2015 | Gm20:8865408 | 20 | 8865408 | 15.83 | -3.11 | -16.62 | 5.24 | 2.32 | 1.48E-16 | SIG |
| FWR | BLUP | Gm01:18303798 | 1 | 18303798 | 5.21 | 0.24 | -3.72 | 0.48 | 2.53 | 6.17E-06 | SUG |
| FWR | BLUP | Gm02:23027268 | 2 | 23027268 | 16.52 | 1.31 |  | 0.61 | 3.20 | 2.70E-18 | SIG |
| FWR | BLUP | Gm02:26772532 | 2 | 26772532 | 10.94 | 1.25 | 0.22 | 0.81 | 4.25 | 1.15E-11 | SIG |
| FWR | BLUP | snp9089 | 2 | 39687479 | 23.08 | 1.61 | -0.13 | 0.71 | 3.73 | 8.35E-24 | SIG |
| FWR | BLUP | snp11062 | 3 | 1445425 | 9.66 | -0.93 | 3.46 | 0.42 | 2.22 | 2.17E-10 | SIG |
| FWR | BLUP | snp16804 | 4 | 6599200 | 4.73 | -0.40 | 2.65 | 0.44 | 2.31 | 1.85E-05 | SUG |
| FWR | BLUP | snp18643 | 4 | 26590103 | 11.50 | 0.98 | -5.20 | 0.29 | 1.53 | 3.16E-12 | SIG |
| FWR | BLUP | snp34353 | 7 | 17960593 | 7.24 | -0.85 | -0.08 | 0.31 | 1.63 | 5.69E-08 | SIG |
| FWR | BLUP | Gm09:45457828 | 9 | 45457828 | 26.88 | -1.94 | -0.09 | 0.58 | 3.04 | 1.32E-27 | SIG |
| FWR | BLUP | snp70623 | 14 | 30110481 | 11.73 | 1.09 | -0.63 | 0.24 | 1.27 | 1.85E-12 | SIG |
| FWR | BLUP | Gm14:45815024 | 14 | 45815024 | 27.16 | 1.80 | -1.28 | 0.90 | 4.76 | 7.01E-28 | SIG |
| FWR | BLUP | Gm15:990450 | 15 | 990450 | 6.71 | 0.14 | -2.57 | 0.63 | 3.31 | 1.94E-07 | SUG |
| FWR | BLUP | snp75353 | 15 | 18451195 | 6.24 | -0.33 | 3.18 | 0.59 | 3.13 | 5.70E-07 | SUG |
| FWR | BLUP | snp78793 | 16 | 791059 | 6.70 | 0.79 | -1.17 | 0.30 | 1.57 | 1.99E-07 | SUG |
| FWR | BLUP | snp79024 | 16 | 2339314 | 7.71 | -0.86 | 0.87 | 0.37 | 1.93 | 1.95E-08 | SIG |
| FWR | BLUP | snp82950 | 16 | 32348668 | 16.02 | -1.28 | 1.68 | 0.44 | 2.34 | 9.67E-17 | SIG |
| FWR | BLUP | snp92434 | 18 | 46392083 | 17.80 | -1.10 | -8.05 | 0.57 | 3.02 | 1.59E-18 | SIG |
| FWR | BLUP | snp96349 | 19 | 5158419 | 3.70 | 0.32 | -4.19 | 0.30 | 1.57 | 2.00E-04 | SUG |
| DWR | 2009 | Gm02:22666322 | 2 | 22666322 | 18.66 | 6.83 |  | 14.36 | 5.69 | 1.85E-20 | SIG |
| DWR | 2009 | snp7912 | 2 | 25984087 | 13.78 | 5.81 | 4.26 | 16.96 | 6.72 | 1.64E-14 | SIG |
| DWR | 2009 | snp31341 | 6 | 50325934 | 29.97 | 7.73 | 33.05 | 16.30 | 6.46 | 1.08E-30 | SIG |
| DWR | 2009 | Gm09:46355897 | 9 | 46355897 | 14.88 | -6.98 | 2.32 | 18.81 | 7.45 | 1.31E-15 | SIG |
| DWR | 2009 | Gm11:11627426 | 11 | 11627426 | 26.17 | -8.38 |  | 9.31 | 3.69 | 4.89E-28 | SIG |
| DWR | 2009 | snp54522 | 11 | 15395305 | 3.04 | 2.54 | -3.53 | 6.42 | 2.54 | 9.14E-04 | SUG |
| DWR | 2009 | snp67755 | 14 | 2766500 | 8.01 | 4.29 | 2.30 | 6.60 | 2.61 | 9.71E-09 | SIG |
| DWR | 2009 | Gm18:58900070 | 18 | 58900070 | 10.11 | -2.75 | -9.02 | 6.78 | 2.69 | 7.78E-11 | SIG |
| DWR | 2009 | snp104710 | 20 | 38027453 | 7.92 | 2.54 | -31.82 | 13.77 | 5.46 | 1.19E-08 | SIG |
| DWR | 2010 | Gm06:3502387 | 6 | 3502387 | 143.73 | 49.29 | 39.77 | 41.22 | 9.39 | 1.89E-144 | SIG |
| DWR | 2010 | Gm11:6095319 | 11 | 6095319 | 6.97 | 5.70 | -0.91 | 6.76 | 1.54 | 1.06E-07 | SUG |
| DWR | 2010 | snp74686 | 15 | 13846003 | 7.59 | -5.56 | 2.62 | 11.95 | 2.72 | 2.58E-08 | SIG |
| DWR | 2010 | snp74923 | 15 | 15356768 | 3.71 | -0.44 | 15.39 | 13.93 | 3.17 | 1.93E-04 | SUG |
| DWR | 2010 | snp76042 | 15 | 23822285 | 6.23 | 4.76 | -9.45 | 18.85 | 4.29 | 5.93E-07 | SUG |
| DWR | 2010 | snp79853 | 16 | 6985532 | 3.85 | -0.35 | -44.45 | 14.52 | 3.31 | 1.42E-04 | SUG |
| DWR | 2010 | snp88210 | 18 | 1505263 | 9.57 | -6.17 | 11.26 | 15.83 | 3.61 | 2.69E-10 | SIG |
| DWR | 2010 | Gm18:37023863 | 18 | 37023863 | 8.12 | 5.07 | -11.64 | 17.25 | 3.93 | 7.52E-09 | SIG |
| DWR | 2010 | snp104312 | 20 | 35749672 | 47.26 | 16.55 |  | 12.17 | 2.77 | 2.99E-49 | SIG |
| DWR | 2014 | snp11901 | 3 | 7062221 | 5.78 | 0.29 | -72.72 | 19.81 | 4.61 | 1.64E-06 | SUG |
| DWR | 2014 | snp24293 | 5 | 32472416 | 14.88 | -5.23 | -61.37 | 25.93 | 6.04 | 1.33E-15 | SIG |
| DWR | 2014 | snp34353 | 7 | 17960593 | 5.13 | -3.92 | 8.84 | 10.49 | 2.44 | 7.43E-06 | SUG |
| DWR | 2014 | Gm07:27138497 | 7 | 27138497 | 4.33 | 0.96 | -12.68 | 14.59 | 3.40 | 4.68E-05 | SUG |
| DWR | 2014 | snp42486 | 8 | 46160353 | 25.88 | 10.41 | -3.66 | 16.48 | 3.84 | 1.34E-26 | SIG |
| DWR | 2014 | snp44761 | 9 | 20036602 | 19.53 | 8.66 | -11.74 | 15.08 | 3.51 | 2.94E-20 | SIG |
| DWR | 2014 | Gm13:27782818 | 13 | 27782818 | 9.03 | 6.48 | 2.48 | 6.75 | 1.57 | 9.43E-10 | SIG |
| DWR | 2014 | snp67365 | 13 | 44155095 | 31.35 | 11.63 |  | 15.68 | 3.65 | 2.96E-33 | SIG |
| DWR | 2014 | Gm14:15466523 | 14 | 15466523 | 6.77 | 3.75 | -12.07 | 16.63 | 3.87 | 1.70E-07 | SUG |
| DWR | 2014 | Gm14:48609310 | 14 | 48609310 | 14.95 | -7.44 | 7.66 | 16.37 | 3.81 | 1.11E-15 | SIG |
| DWR | 2014 | Gm15:30405085 | 15 | 30405085 | 4.26 | 1.40 | -19.31 | 13.93 | 3.24 | 5.46E-05 | SUG |
| DWR | 2014 | Gm18:36550667 | 18 | 36550667 | 5.00 | -2.11 | -22.02 | 10.16 | 2.36 | 9.99E-06 | SUG |
| DWR | 2014 | snp93654 | 18 | 54072015 | 6.76 | -4.92 | -0.46 | 11.74 | 2.73 | 1.73E-07 | SUG |
| DWR | 2014 | Gm19:5374426 | 19 | 5374426 | 13.34 | 7.46 | -1.58 | 18.27 | 4.25 | 4.56E-14 | SIG |
| DWR | 2015 | snp24290 | 5 | 32428997 | 10.01 | -7.79 | 3.35 | 25.00 | 6.06 | 9.67E-11 | SIG |
| DWR | 2015 | Gm13:44246275 | 13 | 44246275 | 4.00 | -1.81 | -19.65 | 15.24 | 3.69 | 9.91E-05 | SUG |
| DWR | BLUP | Gm01:30672463 | 1 | 30672463 | 17.46 | 2.28 | -3.22 | 0.89 | 2.74 | 3.48E-18 | SIG |
| DWR | BLUP | snp24293 | 5 | 32472416 | 15.84 | -1.59 | -17.94 | 2.08 | 6.41 | 1.44E-16 | SIG |
| DWR | BLUP | snp34353 | 7 | 17960593 | 9.21 | -1.64 | 0.46 | 1.21 | 3.75 | 6.13E-10 | SIG |
| DWR | BLUP | snp46515 | 9 | 39388638 | 13.88 | 2.03 | 1.27 | 1.22 | 3.78 | 1.33E-14 | SIG |
| DWR | BLUP | snp55953 | 11 | 31991853 | 8.25 | 1.50 | -3.09 | 1.62 | 4.99 | 5.63E-09 | SIG |
| DWR | BLUP | Gm13:27570933 | 13 | 27570933 | 16.27 | 2.40 | 0.45 | 1.49 | 4.61 | 5.35E-17 | SIG |
| DWR | BLUP | snp68284 | 14 | 5836612 | 12.85 | 1.95 | 1.38 | 0.85 | 2.62 | 1.43E-13 | SIG |
| DWR | BLUP | Gm14:20196896 | 14 | 20196896 | 7.21 | 1.17 | -2.93 | 1.41 | 4.37 | 6.23E-08 | SIG |
| DWR | BLUP | snp74927 | 15 | 15393585 | 10.73 | -1.73 | 2.21 | 2.28 | 7.04 | 1.88E-11 | SIG |
| DWR | BLUP | Gm18:8282900 | 18 | 8282900 | 10.31 | 1.54 | -3.93 | 1.32 | 4.08 | 4.96E-11 | SIG |

DWR: the dry weights of roots; FWR: the fresh weights of roots; LH: the length of hypocotyls; LR: the length of main root; Env: environment; BLUP: best linear unbiased prediction; Chr: chromosome; Pos: position; Add: additive; Dom: dominance; Var: variance; SIG: significant (-log10(*P*) ≥ 7.17); SUG: suggestion (LOD ≥ 3).

**Table S5. QTNs for soybean salt tolerance index traits in all the environments using 3VmrMLM.**

| **Trait** | **Marker** | **Chr** | **Pos (bp)** | **LOD (Q)** | **Add** | **Dom** | **Var** | **r^2^ (%)** | ***P*-value** | **Significance** |
| --- | --- | --- | --- | --- | --- | --- | --- | --- | --- | --- |
| DWR | Gm04:11967582 | 4 | 11967582 | 4.91 | 2.69 | -0.57 | 4.68 | 0.79 | 1.23E-05 | SUG |
| DWR | Gm05:15471311 | 5 | 15471311 | 21.60 | -4.62 | NA | 6.35 | 1.07 | 2.01E-23 | SIG |
| DWR | snp24293 | 5 | 32472416 | 31.05 | -4.80 | -33.80 | 8.05 | 1.36 | 8.83E-32 | SIG |
| DWR | Gm06:26076706 | 6 | 26076706 | 34.60 | -6.07 | 1.99 | 3.43 | 0.58 | 2.50E-35 | SIG |
| DWR | Gm07:1291917 | 7 | 1291917 | 8.76 | 0.81 | -6.35 | 8.06 | 1.36 | 1.75E-09 | SIG |
| DWR | snp37181 | 8 | 3439148 | 5.14 | -4.78 | 1.02 | 4.89 | 0.83 | 7.27E-06 | SUG |
| DWR | snp46475 | 9 | 39073956 | 10.27 | -3.20 | 0.65 | 8.38 | 1.42 | 5.33E-11 | SIG |
| DWR | snp54522 | 11 | 15395305 | 5.98 | 2.41 | -0.60 | 5.42 | 0.92 | 1.05E-06 | SUG |
| DWR | snp73790 | 15 | 8445573 | 7.75 | -2.81 | -0.53 | 5.37 | 0.91 | 1.78E-08 | SIG |
| DWR | Gm17:34244436 | 17 | 34244436 | 8.09 | -2.24 | 4.73 | 6.48 | 1.09 | 8.21E-09 | SIG |
| DWR | Gm17:39312399 | 17 | 39312399 | 4.70 | -2.54 | 0.07 | 4.44 | 0.75 | 2.01E-05 | SUG |
| DWR | snp96645 | 19 | 8047060 | 17.22 | 4.01 | 7.91 | 6.93 | 1.17 | 6.10E-18 | SIG |
| DWR | snp98775 | 19 | 36877474 | 3.28 | 1.80 | 1.29 | 2.61 | 0.44 | 5.29E-04 | SUG |
| DWR | Gm20:43549302 | 20 | 43549302 | 6.02 | -2.77 | 1.65 | 5.49 | 0.93 | 9.53E-07 | SUG |
| FWR | snp11062 | 3 | 1445425 | 12.68 | -2.16 | 0.20 | 1.59 | 0.47 | 2.08E-13 | SIG |
| FWR | Gm03:5020938 | 3 | 5020938 | 5.69 | 1.63 | -0.59 | 2.05 | 0.61 | 2.06E-06 | SUG |
| FWR | snp24293 | 5 | 32472416 | 5.17 | -0.37 | -15.29 | 1.65 | 0.49 | 6.73E-06 | SUG |
| FWR | snp34353 | 7 | 17960593 | 10.47 | -1.97 | 1.35 | 1.80 | 0.53 | 3.40E-11 | SIG |
| FWR | Gm07:30844218 | 7 | 30844218 | 18.88 | 2.50 | -4.41 | 2.67 | 0.79 | 1.33E-19 | SIG |
| FWR | Gm08:29003459 | 8 | 29003459 | 4.94 | -0.13 | -7.49 | 1.65 | 0.49 | 1.15E-05 | SUG |
| FWR | snp51540 | 10 | 44100386 | 5.10 | 1.33 | 1.86 | 1.40 | 0.41 | 7.95E-06 | SUG |
| FWR | Gm12:26356956 | 12 | 26356956 | 5.77 | 1.44 | NA | 1.49 | 0.44 | 2.52E-07 | SUG |
| FWR | Gm13:13405805 | 13 | 13405805 | 33.44 | -2.98 | -10.86 | 2.69 | 0.80 | 3.67E-34 | SIG |
| FWR | Gm15:43667149 | 15 | 43667149 | 23.53 | -1.99 | -6.01 | 2.30 | 0.68 | 2.96E-24 | SIG |
| FWR | snp78166 | 15 | 48224134 | 8.32 | -0.77 | -13.71 | 2.20 | 0.65 | 4.78E-09 | SIG |
| FWR | snp78793 | 16 | 791059 | 21.58 | 2.87 | 0.95 | 2.89 | 0.86 | 2.63E-22 | SIG |
| FWR | snp79214 | 16 | 3530336 | 12.97 | 2.19 | 1.99 | 1.58 | 0.47 | 1.06E-13 | SIG |
| FWR | Gm16:27676766 | 16 | 27676766 | 9.48 | 1.40 | -2.51 | 3.44 | 1.02 | 3.31E-10 | SIG |
| FWR | Gm17:16111682 | 17 | 16111682 | 5.54 | -1.29 | 2.66 | 0.92 | 0.27 | 2.87E-06 | SUG |
| FWR | Gm18:38106844 | 18 | 38106844 | 8.05 | -1.09 | -6.05 | 1.24 | 0.37 | 8.96E-09 | SIG |
| FWR | Gm18:46447482 | 18 | 46447482 | 12.61 | -2.49 | -0.64 | 4.27 | 1.27 | 2.46E-13 | SIG |
| LH | snp151 | 1 | 1285099 | 19.88 | -4.37 | -1.26 | 2.41 | 0.85 | 1.31E-20 | SIG |
| LH | snp1466 | 1 | 11851702 | 29.59 | -1.70 | -15.04 | 7.26 | 2.55 | 2.56E-30 | SIG |
| LH | Gm03:2511712 | 3 | 2511712 | 41.19 | -3.56 | -9.46 | 2.26 | 0.79 | 6.46E-42 | SIG |
| LH | Gm03:33210628 | 3 | 33210628 | 12.46 | 2.66 | -1.32 | 4.83 | 1.70 | 3.50E-13 | SIG |
| LH | snp24404 | 5 | 33480110 | 22.66 | 3.09 | -1.71 | 4.52 | 1.59 | 2.18E-23 | SIG |
| LH | snp25529 | 6 | 587662 | 11.75 | 2.24 | -0.01 | 4.34 | 1.53 | 1.79E-12 | SIG |
| LH | snp30631 | 6 | 45611572 | 11.95 | -2.24 | -1.07 | 4.75 | 1.67 | 1.13E-12 | SIG |
| LH | snp34355 | 7 | 17965274 | 8.41 | 0.87 | -7.28 | 3.14 | 1.10 | 3.86E-09 | SIG |
| LH | snp56491 | 11 | 36449725 | 7.40 | -1.39 | -4.30 | 2.42 | 0.85 | 3.97E-08 | SIG |
| LH | Gm13:29464820 | 13 | 29464820 | 6.82 | 2.01 | -0.45 | 2.71 | 0.95 | 1.50E-07 | SUG |
| LH | snp74927 | 15 | 15393585 | 7.85 | 0.00 | 8.32 | 2.99 | 1.05 | 1.41E-08 | SIG |
| LH | Gm16:27651990 | 16 | 27651990 | 7.89 | -0.03 | -5.73 | 2.81 | 0.99 | 1.28E-08 | SIG |
| LH | Gm18:61636955 | 18 | 61636955 | 4.14 | 1.57 | -0.29 | 1.57 | 0.55 | 7.25E-05 | SUG |
| LH | snp98807 | 19 | 37061537 | 21.96 | 3.02 | -0.45 | 2.24 | 0.79 | 1.11E-22 | SIG |
| LH | snp104483 | 20 | 36688945 | 5.46 | 0.77 | 2.44 | 1.29 | 0.45 | 3.47E-06 | SUG |
| LH | snp105142 | 20 | 40918551 | 17.41 | -2.69 | 1.20 | 2.96 | 1.04 | 3.87E-18 | SIG |
| LR | snp11499 | 3 | 3949961 | 5.26 | 1.31 | 0.31 | 0.65 | 0.16 | 5.56E-06 | SUG |
| LR | Gm04:10922211 | 4 | 10922211 | 3.01 | 0.12 | -3.61 | 0.93 | 0.23 | 9.77E-04 | SUG |
| LR | Gm04:16148273 | 4 | 16148273 | 4.93 | -0.24 | -4.80 | 1.28 | 0.32 | 1.17E-05 | SUG |
| LR | snp24293 | 5 | 32472416 | 11.55 | 1.14 | -18.12 | 2.80 | 0.70 | 2.79E-12 | SIG |
| LR | Gm06:44259955 | 6 | 44259955 | 17.69 | -2.55 | 0.79 | 2.19 | 0.55 | 2.05E-18 | SIG |
| LR | Gm10:13619213 | 10 | 13619213 | 8.10 | -1.87 | -0.64 | 2.50 | 0.63 | 8.04E-09 | SIG |
| LR | Gm10:24084591 | 10 | 24084591 | 21.98 | -2.11 | -9.14 | 1.75 | 0.44 | 1.05E-22 | SIG |
| LR | Gm13:29000149 | 13 | 29000149 | 11.77 | -0.14 | -8.78 | 3.47 | 0.87 | 1.69E-12 | SIG |
| LR | snp68268 | 14 | 5726780 | 4.66 | -1.22 | 1.16 | 1.47 | 0.37 | 2.18E-05 | SUG |
| LR | Gm15:37410624 | 15 | 37410624 | 6.20 | -0.86 | -5.98 | 0.90 | 0.23 | 6.25E-07 | SUG |
| LR | snp79457 | 16 | 4562128 | 7.00 | 1.49 | 1.60 | 1.75 | 0.44 | 9.97E-08 | SUG |
| LR | Gm16:27649088 | 16 | 27649088 | 9.01 | -0.55 | -5.97 | 2.01 | 0.50 | 9.69E-10 | SIG |
| LR | Gm17:21060750 | 17 | 21060750 | 10.00 | -1.99 | 0.51 | 1.11 | 0.28 | 9.98E-11 | SIG |
| LR | Gm19:18202180 | 19 | 18202180 | 4.67 | -0.42 | -5.33 | 1.06 | 0.27 | 2.15E-05 | SUG |
| LR | Gm20:19034852 | 20 | 19034852 | 8.99 | -1.69 | NA | 1.32 | 0.33 | 1.26E-10 | SIG |
| LR | snp105142 | 20 | 40918551 | 19.21 | -2.51 | -2.43 | 2.34 | 0.59 | 6.24E-20 | SIG |

DWR: the dry weights of roots; FWR: the fresh weights of roots; LH: the length of hypocotyls; LR: the length of main root; Env: environment; Chr: chromosome; Pos: position; Add: additive; Dom: dominance; Var: variance; SIG: significant (-log10(*P*) ≥ 7.17); SUG: suggestion (LOD ≥ 3).

**Table S6.** **QEIs for soybean salt tolerance index traits in all the environments using 3VmrMLM**

| **Trait** | **Marker** | **Chr** | **Pos (bp)** | **LOD** | **Var** | **r^2^ (%)** | ***P*-value** | **Significance** |
| --- | --- | --- | --- | --- | --- | --- | --- | --- |
| DWR | Gm01:45703343 | 1 | 45703343 | 17.73 | 17.45 | 2.95 | 1.35E-17 | SIG |
| DWR | snp20887 | 4 | 47545085 | 10.58 | 10.07 | 1.70 | 8.54E-09 | SIG |
| DWR | Gm05:2927036 | 5 | 2927036 | 27.96 | 27.63 | 4.67 | 2.36E-25 | SIG |
| DWR | Gm06:15393059 | 6 | 15393059 | 30.50 | 30.67 | 5.18 | 3.00E-30 | SIG |
| DWR | snp32442 | 7 | 6345877 | 6.84 | 6.49 | 1.10 | 2.02E-05 | SUG |
| DWR | Gm11:6095319 | 11 | 6095319 | 79.79 | 90.73 | 15.32 | 2.76E-76 | SIG |
| DWR | snp57704 | 12 | 4923868 | 28.29 | 29.33 | 4.95 | 1.14E-25 | SIG |
| DWR | Gm13:10766815 | 13 | 10766815 | 10.80 | 10.35 | 1.75 | 9.04E-11 | SIG |
| DWR | snp77585 | 15 | 43916620 | 4.07 | 3.99 | 0.67 | 4.61E-03 | SUG |
| DWR | Gm16:32801235 | 16 | 32801235 | 15.51 | 15.09 | 2.55 | 2.10E-15 | SIG |
| DWR | Gm17:10987875 | 17 | 10987875 | 27.05 | 26.90 | 4.54 | 8.08E-27 | SIG |
| DWR | Gm18:9322792 | 18 | 9322792 | 15.15 | 14.91 | 2.52 | 4.83E-15 | SIG |
| DWR | Gm19:39779130 | 19 | 39779130 | 7.77 | 7.42 | 1.25 | 8.31E-08 | SUG |
| FWR | Gm02:5905123 | 2 | 5905123 | 6.11 | 2.20 | 0.65 | 8.90E-05 | SUG |
| FWR | snp18510 | 4 | 24898203 | 10.37 | 3.90 | 1.16 | 1.32E-08 | SIG |
| FWR | snp19002 | 4 | 33146175 | 34.14 | 13.08 | 3.88 | 2.28E-31 | SIG |
| FWR | Gm06:4443518 | 6 | 4443518 | 5.16 | 1.86 | 0.55 | 2.78E-05 | SUG |
| FWR | snp27881 | 6 | 16034140 | 10.09 | 3.65 | 1.08 | 2.41E-08 | SIG |
| FWR | Gm06:20707182 | 6 | 20707182 | 59.13 | 23.56 | 6.99 | 7.04E-56 | SIG |
| FWR | Gm08:43019004 | 8 | 43019004 | 75.22 | 31.97 | 9.48 | 9.28E-72 | SIG |
| FWR | snp65914 | 13 | 34943684 | 8.26 | 3.04 | 0.90 | 1.11E-06 | SUG |
| FWR | snp72425 | 14 | 48949774 | 5.74 | 1.99 | 0.59 | 1.86E-04 | SUG |
| FWR | Gm16:26218707 | 16 | 26218707 | 26.12 | 9.88 | 2.93 | 1.42E-23 | SIG |
| FWR | Gm16:36788198 | 16 | 36788198 | 33.53 | 12.71 | 3.77 | 9.09E-31 | SIG |
| FWR | Gm18:11951410 | 18 | 11951410 | 25.25 | 9.30 | 2.76 | 4.93E-25 | SIG |
| FWR | Gm18:13909558 | 18 | 13909558 | 9.61 | 3.43 | 1.02 | 1.33E-09 | SIG |
| FWR | snp90590 | 18 | 20016824 | 27.63 | 10.38 | 3.08 | 4.87E-25 | SIG |
| FWR | Gm18:30303366 | 18 | 30303366 | 11.21 | 4.05 | 1.20 | 3.62E-11 | SIG |
| FWR | Gm18:55544217 | 18 | 55544217 | 21.23 | 7.79 | 2.31 | 7.33E-19 | SIG |
| FWR | snp98278 | 19 | 33223669 | 11.13 | 3.97 | 1.18 | 2.66E-09 | SIG |
| FWR | Gm20:25021253 | 20 | 25021253 | 143.89 | 70.82 | 21.00 | 7.19E-140 | SIG |
| LH | Gm01:18435637 | 1 | 18435637 | 22.70 | 9.45 | 3.32 | 1.65E-22 | SIG |
| LH | Gm02:22437759 | 2 | 22437759 | 8.18 | 3.27 | 1.15 | 3.33E-08 | SIG |
| LH | Gm03:3991074 | 3 | 3991074 | 7.09 | 2.82 | 0.99 | 1.23E-05 | SUG |
| LH | Gm04:23372024 | 4 | 23372024 | 13.56 | 5.43 | 1.91 | 1.44E-11 | SIG |
| LH | snp26457 | 6 | 7351313 | 47.72 | 20.57 | 7.23 | 1.18E-44 | SIG |
| LH | Gm06:22078856 | 6 | 22078856 | 37.42 | 16.16 | 5.68 | 4.06E-37 | SIG |
| LH | Gm09:28051574 | 9 | 28051574 | 31.39 | 13.26 | 4.66 | 3.95E-31 | SIG |
| LH | snp57848 | 12 | 5922375 | 10.38 | 4.15 | 1.46 | 1.30E-08 | SIG |
| LH | Gm13:35351832 | 13 | 35351832 | 13.59 | 5.42 | 1.91 | 1.65E-13 | SIG |
| LH | Gm17:30770384 | 17 | 30770384 | 5.94 | 2.36 | 0.83 | 4.94E-06 | SUG |
| LH | snp89310 | 18 | 9301728 | 101.72 | 49.63 | 17.45 | 5.26E-98 | SIG |
| LH | Gm18:55815732 | 18 | 55815732 | 5.83 | 2.31 | 0.81 | 6.35E-06 | SUG |
| LH | snp94870 | 18 | 59336753 | 9.26 | 3.70 | 1.30 | 1.38E-07 | SUG |
| LR | Gm02:2541312 | 2 | 2541312 | 5.38 | 1.66 | 0.42 | 3.78E-04 | SUG |
| LR | snp6021 | 2 | 4448861 | 7.04 | 2.25 | 0.57 | 1.36E-05 | SUG |
| LR | Gm02:15929739 | 2 | 15929739 | 8.50 | 2.72 | 0.68 | 1.62E-08 | SIG |
| LR | Gm03:15036456 | 3 | 15036456 | 19.15 | 6.23 | 1.56 | 5.33E-19 | SIG |
| LR | Gm04:49157380 | 4 | 49157380 | 20.85 | 7.00 | 1.76 | 1.70E-18 | SIG |
| LR | Gm06:31070814 | 6 | 31070814 | 7.15 | 2.24 | 0.56 | 3.31E-07 | SUG |
| LR | Gm06:36080805 | 6 | 36080805 | 14.99 | 4.88 | 1.22 | 6.41E-13 | SIG |
| LR | Gm07:5557012 | 7 | 5557012 | 27.42 | 9.21 | 2.31 | 7.78E-25 | SIG |
| LR | snp48170 | 10 | 4364392 | 240.20 | 130.66 | 32.76 | 9.80E-236 | SIG |
| LR | Gm11:12393272 | 11 | 12393272 | 14.55 | 4.78 | 1.20 | 1.88E-14 | SIG |
| LR | snp56342 | 11 | 35486458 | 26.98 | 9.05 | 2.27 | 2.09E-24 | SIG |
| LR | Gm14:47141670 | 14 | 47141670 | 22.58 | 7.50 | 1.88 | 2.17E-22 | SIG |
| LR | snp81907 | 16 | 27312836 | 88.66 | 33.92 | 8.51 | 4.66E-85 | SIG |
| LR | Gm17:34524617 | 17 | 34524617 | 121.45 | 49.38 | 12.38 | 1.41E-117 | SIG |
| LR | Gm18:44795654 | 18 | 44795654 | 71.92 | 25.97 | 6.51 | 1.69E-68 | SIG |

DWR: the dry weights of roots; FWR: the fresh weights of roots; LH: the length of hypocotyls; LR: the length of main root; Env: environment; Chr: chromosome; Pos: position; SIG: significant (-log10(*P*) ≥ 7.17); SUG: suggestion (LOD ≥ 3).

**Table S7.** **QEIs for soybean salt tolerance related traits between control and salt treatments using 3VmrMLM.**

| **Trait** | **Env** | **Marker** | **Chr** | **Pos (bp)** | **LOD** | **Var** | **r^2^ (%)** | ***P*-value** | **Significance** |
| --- | --- | --- | --- | --- | --- | --- | --- | --- | --- |
| DWR | 2009 | Gm04:7944726 | 4 | 7944726 | 6.59 | 0.00E+00 | 0.31 | 2.55E-07 | SUG |
| DWR | 2009 | Gm04:10966335 | 4 | 10966335 | 306.87 | 4.00E-04 | 69.86 | 1.37E-307 | SIG |
| DWR | 2009 | Gm04:15508689 | 4 | 15508689 | 3.62 | 0.00E+00 | 0.17 | 2.38E-04 | SUG |
| DWR | 2009 | Gm07:22358181 | 7 | 22358181 | 3.86 | 0.00E+00 | 0.18 | 1.39E-04 | SUG |
| DWR | 2009 | snp42438 | 8 | 45894910 | 129.60 | 1.00E-04 | 10.90 | 2.54E-130 | SIG |
| DWR | 2009 | snp54798 | 11 | 17338166 | 22.49 | 0.00E+00 | 1.13 | 3.23E-23 | SIG |
| DWR | 2009 | Gm12:18789045 | 12 | 18789045 | 4.25 | 0.00E+00 | 0.20 | 5.68E-05 | SUG |
| DWR | 2009 | Gm16:18670167 | 16 | 18670167 | 13.64 | 0.00E+00 | 0.66 | 2.29E-14 | SIG |
| DWR | 2009 | Gm16:27042323 | 16 | 27042323 | 8.13 | 0.00E+00 | 0.38 | 7.46E-09 | SIG |
| DWR | 2009 | snp101810 | 20 | 10387529 | 3.02 | 0.00E+00 | 0.14 | 9.64E-04 | SUG |
| DWR | 2009 | Gm20:25005099 | 20 | 25005099 | 28.70 | 0.00E+00 | 1.48 | 1.98E-29 | SIG |
| DWR | 2010 | snp16826 | 4 | 6704221 | 9.09 | 0.00E+00 | 1.58 | 8.09E-10 | SIG |
| DWR | 2010 | snp74450 | 15 | 12641182 | 75.86 | 0.00E+00 | 17.84 | 1.38E-76 | SIG |
| DWR | 2014 | snp11608 | 3 | 4978167 | 3.59 | 0.00E+00 | 0.74 | 4.76E-05 | SUG |
| DWR | 2014 | Gm07:5583782 | 7 | 5583782 | 11.03 | 0.00E+00 | 2.36 | 9.41E-12 | SIG |
| DWR | 2014 | Gm12:10752400 | 12 | 10752400 | 16.39 | 0.00E+00 | 3.59 | 4.05E-17 | SIG |
| DWR | 2014 | snp67358 | 13 | 44108710 | 14.42 | 0.00E+00 | 3.13 | 3.79E-15 | SIG |
| DWR | 2014 | snp73317 | 15 | 5456731 | 4.78 | 0.00E+00 | 1.00 | 1.66E-05 | SUG |
| DWR | 2015 | snp11600 | 3 | 4961017 | 9.04 | 0.00E+00 | 1.77 | 9.22E-10 | SIG |
| DWR | 2015 | Gm05:31331078 | 5 | 31331078 | 11.80 | 0.00E+00 | 2.34 | 1.57E-12 | SIG |
| DWR | 2015 | Gm07:22721860 | 7 | 22721860 | 22.57 | 0.00E+00 | 4.69 | 2.68E-23 | SIG |
| DWR | 2015 | Gm11:28219968 | 11 | 28219968 | 8.66 | 0.00E+00 | 1.70 | 2.72E-10 | SIG |
| DWR | 2015 | snp59964 | 12 | 26059845 | 6.34 | 0.00E+00 | 1.23 | 6.51E-08 | SIG |
| DWR | 2015 | snp105171 | 20 | 41064342 | 3.98 | 0.00E+00 | 0.76 | 1.05E-04 | SUG |
| DWR | 2015 | snp105597 | 20 | 43552043 | 4.18 | 0.00E+00 | 0.80 | 6.59E-05 | SUG |
| DWR | BLUP | snp74 | 1 | 698005 | 10.32 | 0.00E+00 | 0.58 | 4.83E-11 | SIG |
| DWR | BLUP | snp28209 | 6 | 18224459 | 20.91 | 0.00E+00 | 1.23 | 1.25E-21 | SIG |
| DWR | BLUP | Gm09:29881635 | 9 | 29881635 | 171.76 | 0.00E+00 | 20.05 | 1.77E-172 | SIG |
| DWR | BLUP | snp67375 | 13 | 44239858 | 19.65 | 0.00E+00 | 1.15 | 2.24E-20 | SIG |
| DWR | BLUP | snp70590 | 14 | 29637831 | 9.79 | 0.00E+00 | 0.55 | 1.63E-10 | SIG |
| FWR | 2009 | snp68 | 1 | 674489 | 10.97 | 3.00E-04 | 1.06 | 1.08E-11 | SIG |
| FWR | 2009 | Gm01:3239978 | 1 | 3239978 | 15.00 | 4.00E-04 | 1.48 | 9.53E-17 | SIG |
| FWR | 2009 | snp47747 | 10 | 1295089 | 9.71 | 2.00E-04 | 0.93 | 1.97E-10 | SIG |
| FWR | 2009 | Gm13:6733127 | 13 | 6733127 | 11.50 | 3.00E-04 | 1.12 | 3.14E-12 | SIG |
| FWR | 2009 | snp63857 | 13 | 23065368 | 5.94 | 1.00E-04 | 0.56 | 1.15E-06 | SUG |
| FWR | 2009 | snp67375 | 13 | 44239858 | 9.36 | 2.00E-04 | 0.90 | 4.33E-10 | SIG |
| FWR | 2009 | snp82599 | 16 | 30520901 | 6.58 | 2.00E-04 | 0.62 | 2.62E-07 | SUG |
| FWR | 2009 | Gm19:26194965 | 19 | 26194965 | 11.49 | 3.00E-04 | 1.11 | 3.50E-13 | SIG |
| FWR | 2009 | Gm19:39652814 | 19 | 39652814 | 4.41 | 1.00E-04 | 0.42 | 3.86E-05 | SUG |
| FWR | 2010 | Gm01:10159 | 1 | 10159 | 8.55 | 2.00E-04 | 0.80 | 2.83E-09 | SIG |
| FWR | 2010 | snp26262 | 6 | 5690544 | 7.73 | 2.00E-04 | 0.72 | 1.87E-08 | SIG |
| FWR | 2010 | snp28209 | 6 | 18224459 | 8.87 | 2.00E-04 | 0.83 | 1.34E-09 | SIG |
| FWR | 2010 | Gm10:48775867 | 10 | 48775867 | 10.15 | 2.00E-04 | 0.96 | 7.13E-11 | SIG |
| FWR | 2010 | snp74533 | 15 | 13057404 | 18.31 | 4.00E-04 | 1.79 | 4.88E-19 | SIG |
| FWR | 2010 | snp99445 | 19 | 41142913 | 10.73 | 3.00E-04 | 1.02 | 1.88E-11 | SIG |
| FWR | 2014 | Gm01:43454206 | 1 | 43454206 | 19.25 | 4.00E-04 | 2.10 | 4.75E-21 | SIG |
| FWR | 2014 | Gm01:46848682 | 1 | 46848682 | 11.21 | 2.00E-04 | 1.18 | 6.24E-12 | SIG |
| FWR | 2014 | Gm02:33540793 | 2 | 33540793 | 3.92 | 1.00E-04 | 0.40 | 1.21E-04 | SUG |
| FWR | 2014 | snp11608 | 3 | 4978167 | 4.46 | 1.00E-04 | 0.46 | 5.86E-06 | SUG |
| FWR | 2014 | snp27689 | 6 | 14942138 | 11.29 | 2.00E-04 | 1.19 | 5.17E-12 | SIG |
| FWR | 2014 | Gm09:36927289 | 9 | 36927289 | 3.03 | 1.00E-04 | 0.31 | 9.33E-04 | SUG |
| FWR | 2014 | Gm10:35066223 | 10 | 35066223 | 12.00 | 2.00E-04 | 1.27 | 1.06E-13 | SIG |
| FWR | 2014 | Gm12:34834623 | 12 | 34834623 | 3.23 | 1.00E-04 | 0.33 | 5.94E-04 | SUG |
| FWR | 2014 | Gm19:23645655 | 19 | 23645655 | 21.35 | 4.00E-04 | 2.35 | 3.53E-23 | SIG |
| FWR | 2015 | snp10343 | 2 | 48523286 | 18.42 | 5.00E-04 | 1.84 | 3.79E-19 | SIG |
| FWR | 2015 | Gm03:25375325 | 3 | 25375325 | 12.24 | 3.00E-04 | 1.19 | 6.07E-14 | SIG |
| FWR | 2015 | snp32047 | 7 | 4298116 | 9.99 | 2.00E-04 | 0.96 | 1.03E-10 | SIG |
| FWR | 2015 | snp53366 | 11 | 5534742 | 24.06 | 6.00E-04 | 2.46 | 8.76E-25 | SIG |
| FWR | 2015 | snp70596 | 14 | 29652207 | 5.58 | 1.00E-04 | 0.53 | 2.61E-06 | SUG |
| FWR | 2015 | Gm18:26245433 | 18 | 26245433 | 5.44 | 1.00E-04 | 0.51 | 3.67E-06 | SUG |
| FWR | 2015 | Gm19:6850939 | 19 | 6850939 | 13.11 | 3.00E-04 | 1.28 | 7.84E-14 | SIG |
| FWR | 2015 | Gm20:41684133 | 20 | 41684133 | 7.58 | 2.00E-04 | 0.72 | 2.64E-08 | SIG |
| FWR | BLUP | Gm01:46848850 | 1 | 46848850 | 4.68 | 0.00E+00 | 0.19 | 2.08E-05 | SUG |
| FWR | BLUP | snp10343 | 2 | 48523286 | 13.41 | 1.00E-04 | 0.56 | 3.91E-14 | SIG |
| FWR | BLUP | snp17557 | 4 | 12472835 | 15.85 | 1.00E-04 | 0.66 | 1.42E-16 | SIG |
| FWR | BLUP | snp25014 | 5 | 38379318 | 5.36 | 0.00E+00 | 0.21 | 4.36E-06 | SUG |
| FWR | BLUP | snp28209 | 6 | 18224459 | 8.50 | 1.00E-04 | 0.35 | 3.15E-09 | SIG |
| FWR | BLUP | snp36287 | 7 | 41653125 | 36.96 | 3.00E-04 | 1.69 | 1.11E-37 | SIG |
| FWR | BLUP | Gm09:36927289 | 9 | 36927289 | 6.47 | 0.00E+00 | 0.26 | 3.36E-07 | SUG |
| FWR | BLUP | snp67375 | 13 | 44239858 | 14.81 | 1.00E-04 | 0.62 | 1.54E-15 | SIG |
| LH | 2009 | snp5442 | 2 | 394740 | 4.04 | 2.48E-02 | 0.32 | 9.16E-05 | SUG |
| LH | 2009 | Gm03:13944875 | 3 | 13944875 | 6.88 | 4.27E-02 | 0.56 | 1.81E-08 | SIG |
| LH | 2009 | Gm06:34717990 | 6 | 34717990 | 15.31 | 9.86E-02 | 1.29 | 4.87E-16 | SIG |
| LH | 2009 | Gm07:41548361 | 7 | 41548361 | 11.58 | 7.34E-02 | 0.96 | 2.80E-13 | SIG |
| LH | 2009 | snp53788 | 11 | 8398437 | 6.01 | 3.71E-02 | 0.48 | 9.88E-07 | SUG |
| LH | 2009 | Gm13:27660749 | 13 | 27660749 | 31.28 | 2.16E-01 | 2.82 | 5.22E-32 | SIG |
| LH | 2009 | snp77489 | 15 | 43382140 | 6.45 | 4.00E-02 | 0.52 | 3.53E-07 | SUG |
| LH | 2009 | Gm16:35380229 | 16 | 35380229 | 5.05 | 3.11E-02 | 0.41 | 8.86E-06 | SUG |
| LH | 2009 | snp87220 | 17 | 36991049 | 4.09 | 2.50E-02 | 0.33 | 8.22E-05 | SUG |
| LH | 2009 | snp104034 | 20 | 34195100 | 3.47 | 2.12E-02 | 0.28 | 3.38E-04 | SUG |
| LH | 2010 | Gm03:33210745 | 3 | 33210745 | 5.98 | 4.24E-02 | 0.57 | 1.04E-06 | SUG |
| LH | 2010 | Gm06:48913867 | 6 | 48913867 | 9.57 | 6.88E-02 | 0.93 | 2.72E-10 | SIG |
| LH | 2010 | snp46764 | 9 | 41150323 | 4.93 | 3.48E-02 | 0.47 | 1.16E-05 | SUG |
| LH | 2010 | snp51884 | 10 | 46138494 | 13.87 | 1.02E-01 | 1.37 | 1.34E-14 | SIG |
| LH | 2010 | snp56997 | 12 | 644919 | 24.36 | 1.87E-01 | 2.53 | 4.36E-25 | SIG |
| LH | 2010 | snp57179 | 12 | 1726812 | 8.13 | 5.81E-02 | 0.79 | 7.37E-09 | SIG |
| LH | 2010 | snp57845 | 12 | 5895785 | 5.40 | 3.82E-02 | 0.52 | 3.99E-06 | SUG |
| LH | 2010 | snp79189 | 16 | 3368695 | 11.41 | 8.27E-02 | 1.12 | 3.89E-12 | SIG |
| LH | 2010 | snp86122 | 17 | 19580987 | 21.16 | 1.60E-01 | 2.16 | 7.01E-22 | SIG |
| LH | 2010 | Gm20:32535429 | 20 | 32535429 | 13.59 | 9.95E-02 | 1.34 | 2.58E-14 | SIG |
| LH | 2010 | snp105268 | 20 | 41698865 | 8.33 | 5.96E-02 | 0.81 | 4.72E-09 | SIG |
| LH | 2014 | snp5619 | 2 | 1824056 | 9.62 | 5.89E-02 | 0.47 | 2.40E-10 | SIG |
| LH | 2014 | Gm03:6018120 | 3 | 6018120 | 26.12 | 1.72E-01 | 1.38 | 5.52E-28 | SIG |
| LH | 2014 | Gm03:6749401 | 3 | 6749401 | 5.90 | 3.56E-02 | 0.29 | 1.25E-06 | SUG |
| LH | 2014 | snp25539 | 6 | 618782 | 10.18 | 6.25E-02 | 0.50 | 6.61E-11 | SIG |
| LH | 2014 | Gm06:26376915 | 6 | 26376915 | 4.36 | 2.61E-02 | 0.21 | 4.37E-05 | SUG |
| LH | 2014 | Gm08:37544822 | 8 | 37544822 | 7.00 | 4.24E-02 | 0.34 | 1.00E-07 | SUG |
| LH | 2014 | snp42553 | 8 | 46652013 | 13.74 | 8.57E-02 | 0.69 | 1.81E-14 | SIG |
| LH | 2014 | Gm10:14720515 | 10 | 14720515 | 12.24 | 7.59E-02 | 0.61 | 5.97E-14 | SIG |
| LH | 2014 | snp51699 | 10 | 45109333 | 28.70 | 1.91E-01 | 1.53 | 1.98E-29 | SIG |
| LH | 2014 | snp68923 | 14 | 10242342 | 12.70 | 7.88E-02 | 0.63 | 2.00E-13 | SIG |
| LH | 2014 | snp86193 | 17 | 20546887 | 4.48 | 2.69E-02 | 0.22 | 3.33E-05 | SUG |
| LH | 2014 | Gm18:55779305 | 18 | 55779305 | 8.05 | 4.90E-02 | 0.39 | 9.00E-09 | SIG |
| LH | 2014 | snp95446 | 18 | 61682492 | 4.49 | 2.69E-02 | 0.22 | 3.25E-05 | SUG |
| LH | 2015 | snp7052 | 2 | 11503697 | 7.46 | 5.54E-02 | 0.42 | 3.46E-08 | SIG |
| LH | 2015 | snp7468 | 2 | 14958573 | 4.40 | 3.23E-02 | 0.25 | 3.95E-05 | SUG |
| LH | 2015 | snp9763 | 2 | 44325265 | 4.51 | 3.30E-02 | 0.25 | 3.12E-05 | SUG |
| LH | 2015 | snp11827 | 3 | 6716113 | 16.94 | 1.31E-01 | 1.00 | 1.16E-17 | SIG |
| LH | 2015 | Gm04:24048709 | 4 | 24048709 | 9.38 | 7.01E-02 | 0.54 | 4.21E-10 | SIG |
| LH | 2015 | Gm06:31156964 | 6 | 31156964 | 81.02 | 8.29E-01 | 6.35 | 9.71E-82 | SIG |
| LH | 2015 | snp35783 | 7 | 38000804 | 8.33 | 6.20E-02 | 0.48 | 4.67E-09 | SIG |
| LH | 2015 | Gm09:3553566 | 9 | 3553566 | 7.54 | 5.60E-02 | 0.43 | 2.86E-08 | SIG |
| LH | 2015 | snp57993 | 12 | 7068862 | 5.67 | 4.18E-02 | 0.32 | 2.14E-06 | SUG |
| LH | 2015 | Gm12:26193026 | 12 | 26193026 | 16.97 | 1.31E-01 | 1.01 | 9.46E-19 | SIG |
| LH | 2015 | Gm12:28448675 | 12 | 28448675 | 19.36 | 1.51E-01 | 1.16 | 3.65E-21 | SIG |
| LH | 2015 | snp67491 | 14 | 937675 | 19.75 | 1.54E-01 | 1.18 | 1.79E-20 | SIG |
| LH | 2015 | Gm15:19967291 | 15 | 19967291 | 6.41 | 4.74E-02 | 0.36 | 3.86E-07 | SUG |
| LH | 2015 | snp82823 | 16 | 31773546 | 16.76 | 1.29E-01 | 0.99 | 1.74E-17 | SIG |
| LH | 2015 | snp88495 | 18 | 3443263 | 6.61 | 4.89E-02 | 0.37 | 2.47E-07 | SUG |
| LH | 2015 | snp91316 | 18 | 27386631 | 14.60 | 1.12E-01 | 0.86 | 2.54E-15 | SIG |
| LH | 2015 | snp93385 | 18 | 52687266 | 4.06 | 2.97E-02 | 0.23 | 8.71E-05 | SUG |
| LH | BLUP | snp1466 | 1 | 11851702 | 55.91 | 1.30E-01 | 1.58 | 1.24E-56 | SIG |
| LH | BLUP | snp5619 | 2 | 1824056 | 45.83 | 1.02E-01 | 1.24 | 1.49E-46 | SIG |
| LH | BLUP | snp14102 | 3 | 35069996 | 17.28 | 3.41E-02 | 0.42 | 5.22E-18 | SIG |
| LH | BLUP | snp30051 | 6 | 41578960 | 17.18 | 3.39E-02 | 0.41 | 6.55E-18 | SIG |
| LH | BLUP | snp34265 | 7 | 17422130 | 8.97 | 1.71E-02 | 0.21 | 1.08E-09 | SIG |
| LH | BLUP | snp34355 | 7 | 17965274 | 4.08 | 7.60E-03 | 0.09 | 8.41E-05 | SUG |
| LH | BLUP | snp37167 | 8 | 3371532 | 16.35 | 3.21E-02 | 0.39 | 4.52E-17 | SIG |
| LH | BLUP | snp39268 | 8 | 17117978 | 44.32 | 9.79E-02 | 1.19 | 4.79E-45 | SIG |
| LH | BLUP | snp44107 | 9 | 10666330 | 22.95 | 4.64E-02 | 0.57 | 1.12E-23 | SIG |
| LH | BLUP | snp56068 | 11 | 32955274 | 18.61 | 3.69E-02 | 0.45 | 2.46E-19 | SIG |
| LH | BLUP | snp57235 | 12 | 2202736 | 3.95 | 7.40E-03 | 0.09 | 1.13E-04 | SUG |
| LH | BLUP | snp65832 | 13 | 34455812 | 17.18 | 3.39E-02 | 0.41 | 6.59E-18 | SIG |
| LH | BLUP | Gm16:27653357 | 16 | 27653357 | 8.60 | 1.64E-02 | 0.20 | 2.52E-09 | SIG |
| LH | BLUP | Gm18:14372030 | 18 | 14372030 | 16.76 | 3.30E-02 | 0.40 | 1.57E-18 | SIG |
| LH | BLUP | snp91316 | 18 | 27386631 | 8.33 | 1.58E-02 | 0.19 | 4.70E-09 | SIG |
| LR | 2009 | snp74 | 1 | 698005 | 7.12 | 1.16E-01 | 0.85 | 7.55E-08 | SUG |
| LR | 2009 | Gm01:45315521 | 1 | 45315521 | 5.04 | 8.10E-02 | 0.59 | 1.45E-06 | SUG |
| LR | 2009 | Gm04:33003606 | 4 | 33003606 | 12.91 | 2.15E-01 | 1.58 | 1.24E-14 | SIG |
| LR | 2009 | snp36479 | 7 | 42964702 | 15.83 | 2.67E-01 | 1.96 | 1.49E-16 | SIG |
| LR | 2009 | snp41199 | 8 | 38938355 | 14.74 | 2.47E-01 | 1.81 | 1.81E-15 | SIG |
| LR | 2009 | snp63925 | 13 | 23368201 | 5.14 | 8.26E-02 | 0.61 | 7.28E-06 | SUG |
| LR | 2009 | Gm20:21864075 | 20 | 21864075 | 43.14 | 8.22E-01 | 6.03 | 7.21E-44 | SIG |
| LR | 2009 | Gm20:40862289 | 20 | 40862289 | 17.80 | 3.03E-01 | 2.22 | 1.40E-19 | SIG |
| LR | 2010 | snp9588 | 2 | 43179754 | 5.58 | 6.31E-02 | 0.40 | 2.62E-06 | SUG |
| LR | 2010 | snp29676 | 6 | 38625296 | 8.39 | 9.60E-02 | 0.61 | 4.09E-09 | SIG |
| LR | 2010 | Gm09:5657313 | 9 | 5657313 | 6.54 | 7.42E-02 | 0.47 | 2.91E-07 | SUG |
| LR | 2010 | Gm09:32400986 | 9 | 32400986 | 23.85 | 2.92E-01 | 1.86 | 1.08E-25 | SIG |
| LR | 2010 | snp53078 | 11 | 3548679 | 6.82 | 7.75E-02 | 0.49 | 2.10E-08 | SIG |
| LR | 2010 | Gm11:14822339 | 11 | 14822339 | 17.88 | 2.13E-01 | 1.36 | 1.14E-19 | SIG |
| LR | 2010 | Gm11:16827888 | 11 | 16827888 | 22.24 | 2.70E-01 | 1.72 | 4.48E-24 | SIG |
| LR | 2010 | Gm15:35987103 | 15 | 35987103 | 10.44 | 1.21E-01 | 0.77 | 4.06E-12 | SIG |
| LR | 2010 | snp84258 | 17 | 4479708 | 4.44 | 5.00E-02 | 0.32 | 3.62E-05 | SUG |
| LR | 2010 | Gm17:32131195 | 17 | 32131195 | 17.79 | 2.12E-01 | 1.35 | 1.42E-19 | SIG |
| LR | 2014 | Gm11:31047641 | 11 | 31047641 | 4.94 | 1.11E-01 | 0.93 | 1.14E-05 | SUG |
| LR | 2014 | Gm16:13157301 | 16 | 13157301 | 5.35 | 1.20E-01 | 1.00 | 4.48E-06 | SUG |
| LR | 2014 | Gm17:19393689 | 17 | 19393689 | 6.61 | 1.49E-01 | 1.25 | 3.41E-08 | SIG |
| LR | 2014 | Gm17:28333341 | 17 | 28333341 | 22.25 | 5.36E-01 | 4.49 | 4.38E-24 | SIG |
| LR | 2015 | Gm01:14786228 | 1 | 14786228 | 24.54 | 4.42E-01 | 2.23 | 2.87E-25 | SIG |
| LR | 2015 | snp10343 | 2 | 48523286 | 8.27 | 1.39E-01 | 0.70 | 5.33E-09 | SIG |
| LR | 2015 | snp11878 | 3 | 6940158 | 6.44 | 1.07E-01 | 0.54 | 3.65E-07 | SUG |
| LR | 2015 | snp17749 | 4 | 14528114 | 4.36 | 7.20E-02 | 0.36 | 4.38E-05 | SUG |
| LR | 2015 | Gm06:43910854 | 6 | 43910854 | 8.63 | 1.45E-01 | 0.73 | 2.36E-09 | SIG |
| LR | 2015 | Gm07:16209209 | 7 | 16209209 | 14.63 | 2.53E-01 | 1.27 | 2.23E-16 | SIG |
| LR | 2015 | Gm10:11312903 | 10 | 11312903 | 13.36 | 2.29E-01 | 1.16 | 4.35E-15 | SIG |
| LR | 2015 | Gm12:12220096 | 12 | 12220096 | 23.17 | 4.14E-01 | 2.09 | 6.75E-24 | SIG |
| LR | 2015 | Gm12:21565617 | 12 | 21565617 | 27.64 | 5.04E-01 | 2.54 | 2.27E-28 | SIG |
| LR | 2015 | snp77813 | 15 | 46570744 | 13.44 | 2.31E-01 | 1.16 | 3.67E-14 | SIG |
| LR | 2015 | snp80040 | 16 | 7789725 | 5.90 | 9.81E-02 | 0.49 | 1.27E-06 | SUG |
| LR | 2015 | Gm18:26528245 | 18 | 26528245 | 4.53 | 7.49E-02 | 0.38 | 4.93E-06 | SUG |
| LR | BLUP | snp10343 | 2 | 48523286 | 7.19 | 2.03E-02 | 0.19 | 6.49E-08 | SIG |
| LR | BLUP | snp17786 | 4 | 14800652 | 6.93 | 1.96E-02 | 0.19 | 1.18E-07 | SUG |
| LR | BLUP | Gm07:30087591 | 7 | 30087591 | 15.12 | 4.42E-02 | 0.42 | 7.19E-17 | SIG |
| LR | BLUP | snp39017 | 8 | 15590718 | 15.10 | 4.41E-02 | 0.42 | 7.94E-16 | SIG |
| LR | BLUP | snp57993 | 12 | 7068862 | 27.51 | 8.46E-02 | 0.81 | 3.11E-28 | SIG |
| LR | BLUP | snp66073 | 13 | 35982335 | 29.88 | 9.28E-02 | 0.89 | 1.33E-30 | SIG |
| LR | BLUP | Gm15:12650007 | 15 | 12650007 | 14.23 | 4.14E-02 | 0.40 | 5.74E-16 | SIG |
| LR | BLUP | snp82889 | 16 | 32109366 | 5.51 | 1.55E-02 | 0.15 | 3.12E-06 | SUG |
| LR | BLUP | Gm17:21060750 | 17 | 21060750 | 16.61 | 4.88E-02 | 0.47 | 2.45E-17 | SIG |

DWR: the dry weights of roots; FWR: the fresh weights of roots; LH: the length of hypocotyls; LR: the length of main root; Env: environment; Chr: chromosome; Pos: position; SIG: significant (-log10(*P*) ≥ 7.17); SUG: suggestion (LOD ≥ 3).

**Table S8. The KEGG pathways of genes in co-expression modules using KOBAS software**

| **Module** | **KEGG pathway** | **ID** | **Input number** | **Background number** | ***P*-Value** | **Corrected *P*-Value** | **Input** |
| --- | --- | --- | --- | --- | --- | --- | --- |
| turquoise | Metabolic pathways | gmx01100 | 67 | 4144 | 7.29E-12 | 5.11E-10 | Glyma17g07530; Glyma20g28980; Glyma06g19820; Glyma20g38950; Glyma15g25060; Glyma07g37570; Glyma03g41600; Glyma09g33650; Glyma18g06840; Glyma14g37440; Glyma13g11700; Glyma01g32090; Glyma19g02270; Glyma14g34610; Glyma06g13210; Glyma11g27720; Glyma03g29440; Glyma16g27210; Glyma05g31390; Glyma01g02580; Glyma02g06400; Glyma10g00790; Glyma18g02210; Glyma17g15820; Glyma12g10780; Glyma16g02250; Glyma12g10580; Glyma17g04340; Glyma01g24931; Glyma11g27480; Glyma06g08670; Glyma05g29400; Glyma04g41540; Glyma08g12530; Glyma16g01630; Glyma18g52070; Glyma16g29790; Glyma12g06300; Glyma13g05830; Glyma07g30210; Glyma06g12610; Glyma17g04920; Glyma04g19030; Glyma04g39860; Glyma12g30490; Glyma20g28720; Glyma09g08150; Glyma09g37020; Glyma10g24630; Glyma09g32430; Glyma08g06300; Glyma13g01420; Glyma20g18980; Glyma11g04720; Glyma07g05130; Glyma17g06930; Glyma04g03740; Glyma04g14361; Glyma06g45950; Glyma18g43240; Glyma04g41600; Glyma11g01940; Glyma12g03210; Glyma05g36930; Glyma01g43540; Glyma17g13730; Glyma19g24250 |
|  | Biosynthesis of secondary metabolites | gmx01110 | 42 | 2121 | 3.29E-10 | 1.15E-08 | Glyma12g10780; Glyma04g14361; Glyma20g38950; Glyma07g37570; Glyma03g41600; Glyma09g33650; Glyma18g06840; Glyma14g37440; Glyma07g00330; Glyma11g27720; Glyma05g31390; Glyma18g45260; Glyma12g10580; Glyma17g04340; Glyma01g24931; Glyma11g27480; Glyma06g08670; Glyma04g41540; Glyma16g29790; Glyma12g06300; Glyma13g05830; Glyma06g12610; Glyma17g04920; Glyma04g19030; Glyma04g02271; Glyma20g28720; Glyma09g08150; Glyma09g37020; Glyma11g04720; Glyma09g32430; Glyma01g02580; Glyma19g24250; Glyma04g03740; Glyma01g32090; Glyma06g45950; Glyma20g18980; Glyma02g06400; Glyma11g00230; Glyma12g03210; Glyma18g43240; Glyma17g13730; Glyma04g39860 |
|  | Alanine, aspartate and glutamate metabolism | gmx00250 | 9 | 98 | 3.75E-08 | 8.75E-07 | Glyma11g27480; Glyma06g08670; Glyma04g41540; Glyma07g05130; Glyma16g01630; Glyma18g06840; Glyma14g37440; Glyma01g32090; Glyma11g27720 |
|  | Fatty acid degradation | gmx00071 | 7 | 100 | 6.79E-06 | 8.54E-05 | Glyma09g32430; Glyma05g31390; Glyma07g37570; Glyma20g18980; Glyma13g11700; Glyma09g08150; Glyma18g43240 |
|  | Carbon metabolism | gmx01200 | 14 | 492 | 7.08E-06 | 8.54E-05 | Glyma06g08670; Glyma05g31390; Glyma12g10780; Glyma19g24250; Glyma07g05130; Glyma16g01630; Glyma06g45950; Glyma07g30210; Glyma02g06400; Glyma09g33650; Glyma12g10580; Glyma18g43240; Glyma17g13730; Glyma11g04720 |
|  | Biosynthesis of amino acids | gmx01230 | 13 | 426 | 7.32E-06 | 8.54E-05 | Glyma11g27480; Glyma06g08670; Glyma04g41540; Glyma07g05130; Glyma16g01630; Glyma13g05830; Glyma20g38950; Glyma18g06840; Glyma14g37440; Glyma12g10580; Glyma11g27720; Glyma09g37020; Glyma17g04340 |
|  | alpha-Linolenic acid metabolism | gmx00592 | 6 | 97 | 6.00E-05 | 6.00E-04 | Glyma09g32430; Glyma05g31390; Glyma04g03740; Glyma07g37570; Glyma20g18980; Glyma18g43240 |
|  | beta-Alanine metabolism | gmx00410 | 6 | 107 | 1.00E-04 | 7.91E-04 | Glyma05g31390; Glyma07g30210; Glyma04g41600; Glyma09g08150; Glyma06g13210; Glyma18g43240 |
|  | Cysteine and methionine metabolism | gmx00270 | 8 | 211 | 1.02E-04 | 7.91E-04 | Glyma06g08670; Glyma11g04720; Glyma09g37020; Glyma04g41600; Glyma01g32090; Glyma20g28720; Glyma06g13210; Glyma17g04340 |
|  | Fatty acid metabolism | gmx01212 | 6 | 127 | 2.45E-04 | 1.71E-03 | Glyma09g32430; Glyma05g31390; Glyma07g37570; Glyma20g18980; Glyma13g11700; Glyma18g43240 |
|  | 2-Oxocarboxylic acid metabolism | gmx01210 | 5 | 95 | 5.06E-04 | 3.22E-03 | Glyma16g01630; Glyma12g10580; Glyma06g08670; Glyma07g05130; Glyma20g38950 |
|  | Valine, leucine and isoleucine degradation | gmx00280 | 5 | 97 | 5.54E-04 | 3.23E-03 | Glyma01g32090; Glyma09g08150; Glyma20g18980; Glyma07g30210; Glyma06g12610 |
|  | Ubiquitin mediated proteolysis | gmx04120 | 7 | 219 | 7.35E-04 | 3.96E-03 | Glyma17g29800; Glyma14g26660; Glyma05g17900; Glyma06g07800; Glyma11g05670; Glyma17g03610; Glyma06g12640 |
|  | Glutathione metabolism | gmx00480 | 6 | 170 | 1.07E-03 | 5.36E-03 | Glyma05g29400; Glyma08g12530; Glyma19g24250; Glyma04g41600; Glyma06g13210; Glyma16g27210 |
|  | Propanoate metabolism | gmx00640 | 4 | 68 | 1.26E-03 | 5.89E-03 | Glyma05g31390; Glyma18g43240; Glyma07g30210; Glyma06g12610 |
|  | Carbon fixation in photosynthetic organisms | gmx00710 | 5 | 127 | 1.76E-03 | 7.49E-03 | Glyma16g01630; Glyma06g08670; Glyma09g33650; Glyma07g05130; Glyma11g04720 |
|  | Glyoxylate and dicarboxylate metabolism | gmx00630 | 5 | 128 | 1.82E-03 | 7.49E-03 | Glyma12g10780; Glyma12g10580; Glyma06g45950; Glyma17g13730; Glyma11g04720 |
|  | Biosynthesis of unsaturated fatty acids | gmx01040 | 3 | 39 | 2.57E-03 | 1.00E-02 | Glyma05g31390; Glyma18g43240; Glyma20g18980 |
|  | Peroxisome | gmx04146 | 5 | 151 | 3.62E-03 | 1.33E-02 | Glyma13g11700; Glyma05g31390; Glyma20g18980; Glyma20g02150; Glyma18g43240 |
|  | Citrate cycle (TCA cycle) | gmx00020 | 4 | 102 | 5.14E-03 | 1.80E-02 | Glyma02g06400; Glyma12g10580; Glyma09g33650; Glyma11g04720 |
|  | Arginine biosynthesis | gmx00220 | 3 | 58 | 7.37E-03 | 2.40E-02 | Glyma16g01630; Glyma06g08670; Glyma07g05130 |
|  | Pyruvate metabolism | gmx00620 | 5 | 181 | 7.54E-03 | 2.40E-02 | Glyma20g38950; Glyma09g08150; Glyma09g33650; Glyma17g13730; Glyma11g04720 |
|  | **Arginine and proline metabolism** | gmx00330 | 4 | 124 | 9.88E-03 | 3.01E-02 | Glyma06g08670; Glyma04g41600; Glyma09g08150; Glyma06g13210 |
|  | Autophagy - other | gmx04136 | 3 | 67 | 1.07E-02 | 3.13E-02 | Glyma09g00630; Glyma11g03460; Glyma01g41910 |
| magenta | **Plant hormone signal transduction** | gmx04075 | 10 | 675 | 9.60E-08 | 9.60E-07 | Glyma13g01150; Glyma11g04130; Glyma07g04630; Glyma01g41290; Glyma15g19840; Glyma09g08290; Glyma13g17180; Glyma16g01220; Glyma13g17640; Glyma19g44805 |
|  | Plant-pathogen interaction | gmx04626 | 4 | 336 | 1.84E-03 | 9.19E-03 | Glyma03g05220; Glyma02g41300; Glyma02g39870; Glyma14g38010 |
|  | **MAPK signaling pathway** | gmx04016 | 3 | 307 | 1.19E-02 | 3.97E-02 | Glyma03g05220; Glyma02g39870; Glyma14g38010 |
| blue | Ribosome | gmx03010 | 116 | 592 | 2.77E-146 | 1.38E-144 | Glyma07g04890; Glyma03g35540; Glyma01g41620; Glyma14g06170; Glyma03g32380; Glyma16g10700; Glyma01g05740; Glyma05g01180; Glyma05g35030; Glyma05g27940; Glyma11g20570; Glyma05g24930; Glyma04g01270; Glyma11g02190; Glyma18g51660; Glyma18g32680; Glyma10g43770; Glyma04g40470; Glyma05g02570; Glyma07g06590; Glyma20g35000; Glyma15g13650; Glyma02g42260; Glyma10g36610; Glyma07g00700; Glyma15g07420; Glyma15g17010; Glyma13g44690; Glyma19g37370; Glyma02g09370; Glyma19g03520; Glyma02g07420; Glyma02g36070; Glyma04g39940; Glyma04g16660; Glyma02g38450; Glyma07g01540; Glyma20g30970; Glyma03g25520; Glyma11g25910; Glyma15g10220; Glyma08g20960; Glyma08g28800; Glyma02g05540; Glyma03g36560; Glyma04g36140; Glyma05g04670; Glyma08g21960; Glyma12g08050; Glyma08g46070; Glyma20g02170; Glyma13g06920; Glyma10g37840; Glyma07g37060; Glyma09g05030; Glyma15g00610; Glyma10g06040; Glyma16g23730; Glyma04g40720; Glyma10g07680; Glyma02g11540; Glyma14g06630; Glyma16g05090; Glyma20g30810; Glyma20g29981; Glyma13g39490; Glyma08g03480; Glyma03g29810; Glyma14g36620; Glyma13g42090; Glyma02g43080; Glyma07g34440; Glyma05g30780; Glyma15g15800; Glyma11g05160; Glyma07g02270; Glyma08g13970; Glyma07g06580; Glyma08g02070; Glyma18g02970; Glyma01g45060; Glyma10g29170; Glyma01g43110; Glyma13g20010; Glyma11g00450; Glyma02g02140; Glyma05g28880; Glyma03g40530; Glyma10g32580; Glyma13g37610; Glyma0169s00200; Glyma18g01110; Glyma10g00680; Glyma08g45770; Glyma19g44916; Glyma05g34570; Glyma03g21710; Glyma20g30730; Glyma19g39240; Glyma12g30800; Glyma03g33530; Glyma15g23220; Glyma11g11040; Glyma01g00740; Glyma10g08910; Glyma01g03570; Glyma01g03180; Glyma17g11430; Glyma03g37460; Glyma12g03230; Glyma05g26290; Glyma10g02270; Glyma05g27570; Glyma14g38950; Glyma03g40110; Glyma09g02790 |
|  | Protein processing in endoplasmic reticulum | gmx04141 | 10 | 375 | 2.40E-05 | 6.00E-04 | Glyma05g36620; Glyma08g02940; Glyma19g35560; Glyma02g47210; Glyma18g08220; Glyma03g32850; Glyma12g12600; Glyma03g27030; Glyma08g44590; Glyma14g01530 |
| purple | Metabolic pathways | gmx01100 | 11 | 4144 | 1.87E-03 | 3.37E-02 | Glyma13g20170; Glyma19g40041; Glyma10g31590; Glyma10g33650; Glyma06g15030; Glyma15g07710; Glyma15g05820; Glyma10g37200; Glyma03g37441; Glyma01g01180; Glyma09g30370 |
|  | Phenylpropanoid biosynthesis | gmx00940 | 3 | 349 | 4.90E-03 | 4.41E-02 | Glyma13g20170; Glyma06g15030; Glyma15g05820 |

**Table S9. The ANOVA for soybean salt tolerance index traits among different evolution types**

| **Trait** | **Env** | **Between wild and landrace soybeans** | | |  | **Between landrace and improved soybeans** | | |
| --- | --- | --- | --- | --- | --- | --- | --- | --- |
|  |  | **Df** | **F value** | **P value** |  | **Df** | **F value** | **P value** |
| LR | 2009 | 155 | 24.69 | 1.76E-06^***^ |  | 248 | 2.75 | 9.87E-02^ns^ |
|  | 2010 | 155 | 28.19 | 3.76E-07^***^ |  | 259 | 1.46 | 2.28E-01^ns^ |
|  | 2014 | 154 | 32.37 | 6.25E-08^***^ |  | 257 | 1.90 | 1.69E-01^ns^ |
|  | 2015 | 161 | 64.98 | 1.62E-13^***^ |  | 263 | 0.79 | 3.76E-01^ns^ |
|  | BLUP | 165 | 102.55 | 4.76E-19^***^ |  | 270 | 0.79 | 3.76E-01^ns^ |
| LH | 2009 | 155 | 3.23 | 7.43E-02^ns^ |  | 248 | 7.01 | 8.64E-03^**^ |
|  | 2010 | 155 | 6.43 | 1.22E-02^*^ |  | 259 | 0.62 | 4.32E-01^ns^ |
|  | 2014 | 155 | 51.72 | 2.56E-11^***^ |  | 258 | 2.30 | 1.30E-01^ns^ |
|  | 2015 | 161 | 36.93 | 8.56E-09^***^ |  | 263 | 8.02 | 4.97E-03^**^ |
|  | BLUP | 165 | 35.54 | 1.47E-08^***^ |  | 270 | 0.01 | 9.25E-01^ns^ |
| FWR | 2009 | 155 | 0.11 | 7.37E-01^ns^ |  | 248 | 42.61 | 3.74E-10^***^ |
|  | 2010 | 154 | 0.70 | 4.05E-01^ns^ |  | 259 | 23.72 | 1.94E-06^***^ |
|  | 2014 | 153 | 11.06 | 1.10E-03^**^ |  | 256 | 0.03 | 8.62E-01^ns^ |
|  | 2015 | 161 | 22.43 | 4.74E-06^***^ |  | 263 | 9.35 | 2.46E-03^**^ |
|  | BLUP | 165 | 15.91 | 9.99E-05^***^ |  | 270 | 37.13 | 3.80E-09^***^ |
| DWR | 2009 | 153 | 1.42 | 2.36E-01^ns^ |  | 246 | 38.64 | 2.17E-09^***^ |
|  | 2010 | 152 | 3.33 | 6.99E-02^ns^ |  | 257 | 9.20 | 2.67E-03^**^ |
|  | 2014 | 151 | 0.23 | 6.33E-01^ns^ |  | 255 | 0.86 | 3.54E-01^ns^ |
|  | 2015 | 161 | 15.93 | 9.94E-05 |  | 263 | 19.35 | 1.58E-05 |
|  | BLUP | 165 | 3.48 | 6.40E-02^ns^ |  | 270 | 31.34 | 5.31E-08 |

DWR: the dry weights of roots; FWR: the fresh weights of roots; LH: the length of hypocotyls; LR: the length of main root; DF: degree of freedom; *, **, and ***: significant at 0.05, 0.01, and 0.001 level; ns: not significant.
